# Dynamic changes in host gene expression associated with H5N8 avian influenza virus infection in mice

**DOI:** 10.1038/srep16512

**Published:** 2015-11-18

**Authors:** Su-Jin Park, Mukesh Kumar, Hyeok-il Kwon, Rak-Kyun Seong, Kyudong Han, Jae-min Song, Chul-Joong Kim, Young-Ki Choi, Ok Sarah Shin

**Affiliations:** 1 College of Medicine and Medical Research Institute, Chungbuk National University, Cheongju 361-763, Republic of Korea; 2 Department of Tropical Medicine, Medical Microbiology and Pharmacology, Pacific Center for Emerging Infectious Diseases Research, John A. Burns School of Medicine, University of Hawaii at Manoa, Honolulu, HI, 96822, USA; 3 Department of Biomedical Sciences, College of Medicine, Korea University Guro Hospital, Seoul, 152-703, Republic of Korea; 4 Department of Nanobiomedical Science, Dankook University, Cheonan, 330-714 Republic of Korea; 5 Department of Global Medical Science, Sungshin Women’s University, Seoul, 136-742 Republic of Korea; 6 College of Veterinary Medicine, Chungnam National University, Daejeon, 305-764 Republic of Korea; 7 Department of Microbiology, College of Medicine, Korea University, Seoul, 136-701 Republic of Korea

## Abstract

Emerging outbreaks of newly found, highly pathogenic avian influenza (HPAI) A(H5N8) viruses have been reported globally. Previous studies have indicated that H5N8 pathogenicity in mice is relatively moderate compared with H5N1 pathogenicity. However, detailed mechanisms underlying avian influenza pathogenicity are still undetermined. We used a high-throughput RNA-seq method to analyse host and pathogen transcriptomes in the lungs of mice infected with A/MD/Korea/W452/2014 (H5N8) and A/EM/Korea/W149/2006 (H5N1) viruses. Sequenced numbers of viral transcripts and expression levels of host immune-related genes at 1 day post infection (dpi) were higher in H5N8-infected than H5N1-infected mice. Dual sequencing of viral transcripts revealed that in contrast to the observations at 1 dpi, higher number of H5N1 genes than H5N8 genes was sequenced at 3 and 7 dpi, which is consistent with higher viral titres and virulence observed in infected lungs *in vivo*. Ingenuity pathway analysis revealed a more significant upregulation of death receptor signalling, driven by H5N1 than with H5N8 infection at 3 and 7 dpi. Early induction of immune response-related genes may elicit protection in H5N8-infected mice, which correlates with moderate pathogenicity *in vivo*. Collectively, our data provide new insight into the underlying mechanisms of the differential pathogenicity of avian influenza viruses.

The continuing spread of highly pathogenic avian influenza virus (HPAI) among wild birds and poultry is alarming and requires urgent active surveillance; it attracts public concern owing to its potential to become pandemic. HPAI A(H5N1) viruses have been discretely involved in reassortment events that have either generated H5 strains with novel viral neuraminidase (NA) subtypes or donated gene components to other co-circulating strains^1^. The first isolation of A(H5N8) viruses from animals was reported in China in December 2013^2^,^3^,^4^. In the following year, the viral strain BDk/Gochang1(H5N8), genotypically identical to an isolated strain from China, was identified as a reassortment between HPAI A(H5N8)-like and A(H11N9)-like strains^5^,^6^. Owing to the potential for avian influenza viruses circulating in poultry to become transmissible between species and to directly infect humans, more detailed studies on the characterisation of the pathogenesis of H5N8 virus infection are necessary.

Our previous work demonstrated that the MDk/W452(H5N8) strain is less pathogenic than is the EM/W149(H5N1) strain in BALB/c mice^7^. In addition, MDk/W452(H5N8) was able to replicate in human nasal epithelium and lung tissues, although its replication efficiency was less than that of EM/W149(H5N1). Analysis of mouse bronchial alveolar fluids indicated that MDk/W452(H5N8) infection moderately upregulated the expression of cytokines/chemokines, whereas the HPAI A(H5N1) viruses, particularly EM/W149(H5N1), induced robust expression of all the pro-inflammatory cytokines/chemokines, including tumour necrosis factor-α (TNF-α), interleukin-1β (IL-1β), IL-6, regulated on activation, normal T cell expressed and secreted (RANTES), and granulocyte macrophage colony-stimulating factor (GM-CSF). Consistent with this finding, persistent activation and recruitment of immune cells (e.g. T cells, macrophages, and monocytes), the hallmark of severely lethal HPAI A(H5N1) virus infection, was not observed with A(H5N8). Thus, in comparison with H5N1-infected mice, H5N8-infected mice showed moderate pathogenicity, although detailed mechanisms of pathogenicity are yet to be determined.

A better understanding of the global gene changes underlying the multi-step progression of pathogenicity during infection could help develop potential therapeutic strategies for emerging infectious diseases^8^. Very recently, high-throughput RNA sequencing (RNA-seq) technology, which is a powerful way to profile the transcriptome with great efficiency and higher accuracy, has been employed in various viral infections and diseases^9^,^10^,^11^,^12^,^13^,^14^. RNA-seq technology has the potential to reveal the alteration dynamics of the pathogen genome itself and the systemic change in host gene expression in the process of infection by pathogens, which could help uncover the pathogenesis of the infection and interaction mechanism of pathogens.

In the present study, we used the RNA-seq technology to comprehensively annotate dual host/pathogen transcriptomes following infection of mice with H5N8 and H5N1. Our work provides a global view of virus type-specific and post-infection time-specific mRNA profiles, which illustrate the role and mechanisms of viral replication efficiency and host response during avian influenza infection of mice.

## Results

### Analysis of the high-throughput sequencing RNA-seq transcriptome

To gain global and dynamic gene expression profiles of A/MD/Korea/W452/2014- (452, H5N8)- and A/EM/Korea/W149/2006- (149, H5N1)-infected BALB/c mice, RNA-seq was performed to explore the transcriptomes from mouse lungs derived at 1, 3, and 7 day post infection (dpi). H5N1 was used to compare the pathogenicity with recently isolated H5N8 virus. More than 37 million 100-bp paired-end reads were generated. The base quality of RNA-seq reads was checked and analysed using an Agilent RNA 6000 Nano Kit (Supplementary Fig. 1a). Each sample had relatively high sequencing coverage range, as shown by pie charts (Supplementary Fig. 1b). The uniquely mapped reads covered over 80% of the total genomic bases (Supplementary Fig. 1c). The read depth seemed to be distributed relatively evenly along the whole body of the genes, reflecting no introduction of obvious bias during randomly primed reverse transcription and subsequent RNA sequencing.

**Figure 1:**
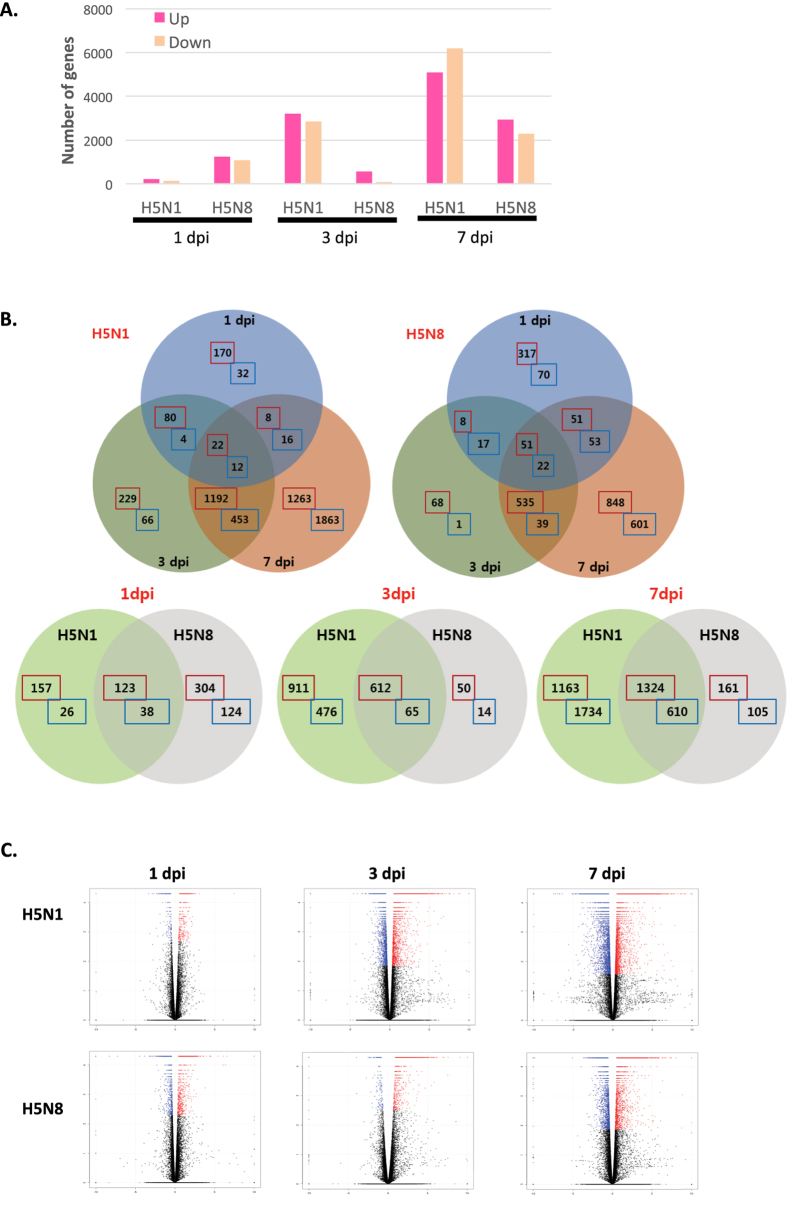
Global overview of the RNA-seq data of H5N8- and H5N1-infected mice lungs. A global overview of RNA-seq data of mice infected with mock, H5N8 virus, or H5N1 virus is shown. Lungs from three mice per group were harvested at 1, 3, and 7 days post infection (dpi). RNA-seq was performed for 21 samples. (**A**) Numbers of more than 2 fold up or down-regulated differentially expressed genes (DEGs) identified from the comparison among mock and virus-infected groups (DEGs were identified based on a false discovery rate (FDR) *q*-value threshold of less than 0.05). (**B**) Venn diagrams of overlapping DEG profiles for H5N8 and H5N1. DEGs are those displaying a change of more than two-fold with a *p*-value of less than or equal to 0.05. The mRNA differential expressions in H5N8- and H5N1-infected mice are depicted in three overlapping circles for 2-fold up and down-regulation at 1, 3, and 7 dpi. The numbers indicate the mRNA counts in the indicated area. (**C**) Volcano plot showing DEGs for H5N1- and H5N8-infected mice. The x-axis represents the log_2_ values of the fold change observed for each mRNA transcript, and the y-axis represents the log_10_ values of the *p*-values of the significance tests between replicates for each transcript. Data for genes that were not classified as differentially expressed are plotted in black.

Using the 41,388 annotated genes in the mouse genome database, gene expression was quantified and compared between the control and the virus-infected groups, and differentially expressed genes (DEGs) with a *q*-value < 0.05 were identified. Figure 1A shows total numbers of both up- and downregulated DEGs for H5N8- and H5N1-infected mice (with +/− 2-fold changes). Interestingly, the number of upregulated genes was larger than the numbers of downregulated genes in both groups of virus-infected mice at both time points, except H5N1 at 7 dpi (Fig. 1A). Furthermore, Venn diagrams were generated to examine the overlapping mRNA profiles for H5N8- and H5N1-infected mice. mRNAs that were up and down regulated at least 2 fold at each time point were used to generate the Venn diagrams. The mRNA differential expression levels in H5N8- and H5N1-infected mice are depicted as three overlapping circles, as shown in Fig. 1A. The numbers indicate the mRNA counts in the indicated area. At 1 dpi, only 3 genes were commonly overlapping upregulated in H5N8- and H5N1-infected mice (Fig. 1B). While higher numbers of genes were upregulated in the lungs of H5N8-infected mice than in those of H5N1-infected mice at 1 dpi, the opposite pattern was observed at 3 and 7 dpi. In H5N1-infected mice, 384 and 760 genes were upregulated more than 2-fold at 3 and 7 dpi, respectively, whereas only 57 and 129 genes were upregulated at 3 and 7 dpi, respectively, in H5N8-infected mice. Overall, there were 245 genes from H5N1 and 492 genes from H5N8 overlapping at 3 and 7 dpi, respectively.

To illustrate that the general expression pattern of transcripts was similarly distributed across all individual biological samples within the group, a volcano plot was generated (Fig. 1C). The ratio of the differential expression (fold change, illustrated in the abscissa of the volcano plot) shows that there was very good correlation between the fold change differences and *p*-values (i.e. genes with a large fold change difference also had a low *p*-value in the group-wise comparison). Additionally, further analysis of DEGs were visualized by hierarchical clustering analysis, scatter plot and MA plot (Supplementary Fig. 2a–c). These data indicate a good clustering of samples according to levels of similarities in the gene expression pattern and there is a clear distinction between these H5N8- and H5N1-infected mice, and the gene expression profiles were able to discriminate between the two main groups of virus-infected mice. To evaluate the degree of dissimilarity among samples in regard to biological variations and dimensions, we then compared H5N8- and H5N1-infected mouse transcriptomes obtained from RNA-seq by performing three-dimensional multidimensional scaling (3D-MDS) (Supplementary Fig. 3). Overall, H5N1 and H5N8 samples at 1 dpi were distinctly distributed from H5N1 and H5N8 samples at 3 and 7 dpi in the 3D-MDS plot, suggesting that these samples are closely clustered according to virus types and post infection time points.

### Viral transcript levels correlate with changes in host gene expression

**Figure 2:**
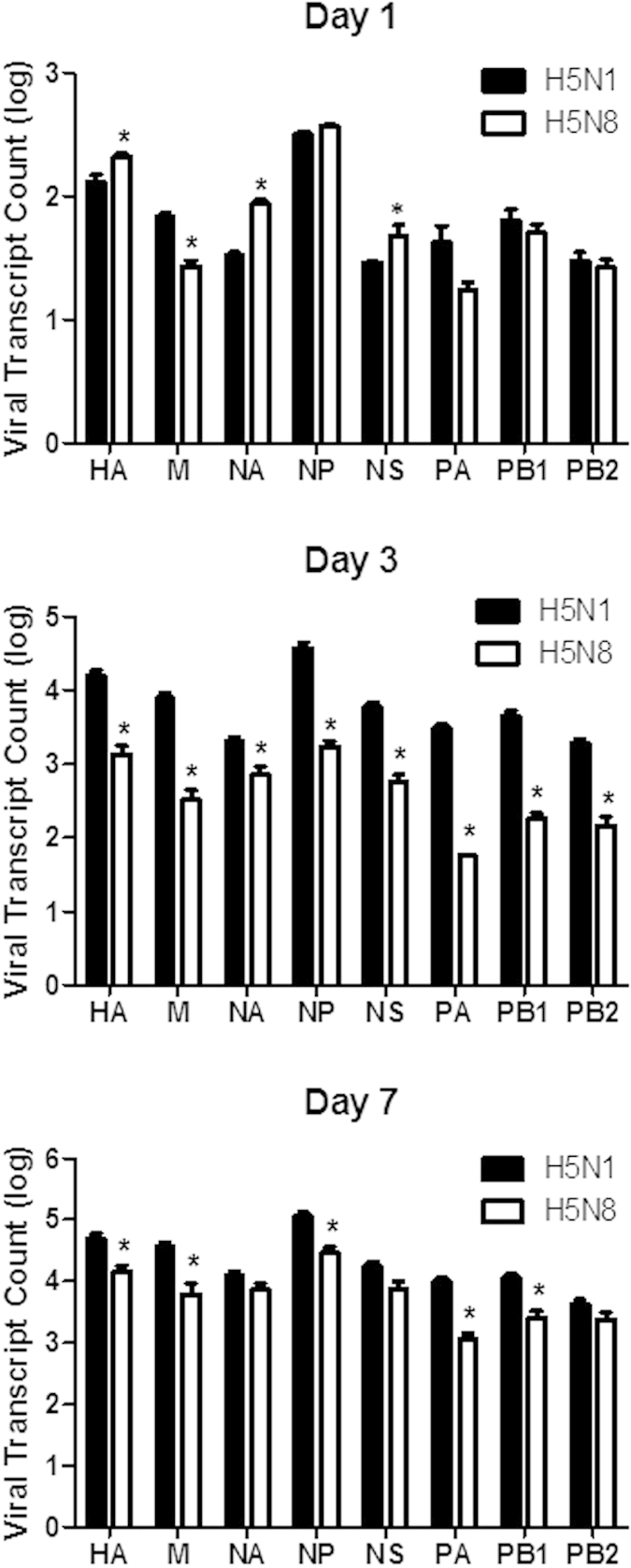
Analysis of viral gene expression changes in the lungs of H5N8- and H5N1-infected mice. Viral gene transcript reads of NA, NP, HA, M, PA, PB1, PB2, and NS are shown. The graph shows mean ± standard deviation of values from three mice per group. Statistical analysis: *p < 0.05 comparing H5N1 and H5N8.

RNA-seq enabled dual analysis of both host and viral transcripts within the same sample. Paired-end reads from influenza-infected samples were mapped to the influenza virus genome. Interestingly, viral transcript counts of hemagglutinin (HA), neuraminidase (NA), and nonstructural protein 1 (NS1) of H5N8 were found to be significantly higher than those of H5N1 at 1 dpi. Given that host transcriptome data also showed more upregulated DEGs for H5N8 in comparison with H5N1 at 1 dpi, these data imply a strong correlation between sequenced numbers of viral transcripts and expression levels of host immune-related genes. Overall, the pattern of influenza virus gene expression was different between H5N8- and H5N1-infected mice in a time-dependent manner. The average number of reads was approximately 10 to 100 times higher in H5N1-infected mice than in H5N8-infected mice at 3 and 7 dpi (Fig. 2). Significantly higher viral expression was observed in the H5N1 group compared with the H5N8 group for every viral gene at 3 dpi (*p* < 0.05). At 7 dpi, the transcript counts for HA, M, NP, PA, and PB1 were found to be significantly different between the H5N1- and H5N8-infected mice. These results suggest that viral gene expression levels also vary for these avian influenza viruses in a temporal dependent manner.

### Distinct and dynamic changes in host DEGs in H5N8- *vs* H5N1-infected mice

To further analyse the characteristics of DEGs, we focused on the 10 DEGs showing the most marked up or down-regulation from H5N8- and H5N1-infected mice (Table 1 and 2). At 1 dpi, there was no significantly upregulated immune-related gene in H5N1-infected mice. However, elevated upregulation of a key immune modulator, chemokine (C-X-C motif) ligand 3 (CXCL-3) was detected in H5N8-infected mice. Interestingly, in contrast to that observed at 1 dpi, the number of DEGs of immune-related function increased dramatically at 3 and 7 dpi. Chemokine pathway-associated genes, such as C-X-C motif chemokine 10 (CXCL-10), CXCL-9, chemokine (C-C motif) ligand 7 (CCL-7), and CXCL-2, were among the 10 most upregulated genes for both H5N8- and H5N1-infected mice at 3 dpi. Furthermore, at 7 dpi, CXCL-11 was found to be one of the most upregulated genes in the H5N8- and H5N1-infected mice, although its expression was higher in H5N1-infected mice than in H5N8-infected mice (10.4 *vs* 8.76 log_2_-fold change (fc) values). We also determined DEGs that were upregulated in virus-infected groups only but not in mock-infected groups. Among those genes, IL-24, which is known to be an important regulator of tumorigenesis^15^, was found to be highly upregulated in the H5N8- (4.87 log_2_ fc values; Supplementary Table 1) and H5N1-infected mice (5.98 log_2_ fc values; Supplementary Table 2). Furthermore, IL-21 and CCL1 were also commonly found to be upregulated in the H5N8- and H5N1-infected mice, but not in the mock-infected group.

To examine the biological roles of the DEGs, a gene ontology (GO) enrichment analysis was applied to the up-regulated genes. GO term statistical analysis of genes in this cluster confirmed that defence response to virus were significantly over-represented (Supplementary Table 3 and 4). Among the biological process category of GO, we focused primarily on immune system processes because many genes related to a function of anti-viral signalling are included in this GO category (Supplementary Fig. 4). Interestingly, in comparison with H5N8-infected mice, higher numbers of genes were found to be included in the GO term of immune system processes in the H5N1-infected mice at all times.

### Comprehensive analysis of functional enrichment and networks in H5N8- *vs* H5N1-infected mice

**Figure 3:**
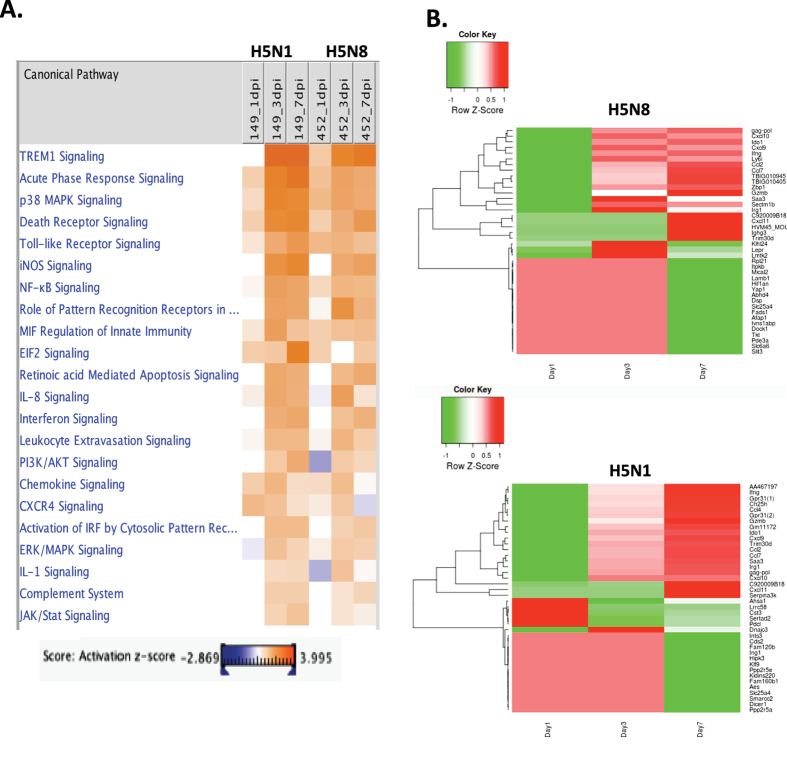
Top canonical signalling pathways activated by H5N8 and H5N1 infections. (**A**) The Ingenuity pathway analysis (IPA) Tool was used to generate a list of the most significant canonical pathways and the highest activated networks with their respective scores obtained from IPA; 149 (H5N1) and 452 (H5N8). (**B**) Heatmaps showing the statistical over-representation of the top 20 up or down-regulated DEGs based on the lists of transcripts found as differentially expressed (compared to the mock-infected conditions).

To investigate possible biological interactions of DEGs and identify important functional networks, datasets representing genes with altered expression profiles derived from RNA-seq analyses were imported into the Ingenuity pathway analysis (IPA) tool. The highest activated networks (high z-score) were identified using IPA. Figure 3A represents a hierarchical clustering heatmap showing the list of activated canonical pathways after influenza virus infection. Notably, triggering receptor expressed on myeloid cells 1 (TREM1) signalling was most activated at 3 and 7 dpi in both H5N8- and H5N1-infected mice. TREM1 is known to activate neutrophils and monocytes/macrophages by signalling through the 12-kDa adapter protein DNAX activation protein (DAP12), and it activates toll-like receptor (TLR) signalling pathways via secretion of proinflammatory cytokines and chemokines in response to microbial infection^16^. Similarly, canonical pathway analysis identified several key innate immune receptor pathways for virus sensing, such as death receptor signalling, toll-like receptor signalling, roles of pattern recognition receptors in virus sensing, retinoic acid-mediated apoptosis signalling, and activation of interferon regulatory factor (IRF) by cytosolic pattern receptors. The heat map also indicates higher activation of death receptor signalling in H5N1-infected mice than in H5N8-infected mice at 3 dpi (z-scores were 4.73 and 3.30 for H5N1 and H5N8, respectively).

Figure 3B also shows the heatmap of the transcriptomic expression values, restricted to the lists of the top 20 up and downregulated DEGs, found as specific for each viral strain. For each set of specific transcripts, hierarchical clustering have been performed and represented using dendrograms. As with the top 10 upregulated gene lists in Table 1, heatmap also identified the chemokine signalling pathway as a key pathway involving the majority of DEGs (CXCL-11, CCL7, CXCL-10, CXCL-2, and CXCL-9) across all datasets, based on the number of network interactions. In addition to virus-sensing pathways, key players in innate immunity and inflammation, such as p38 MAPK signalling, and iNOS signalling, NF-κB signalling, IL-8 signalling, IL-1 signalling, and leukocyte extravasation signalling, were also found to be key pathways involving the majority of DEGs in both H5N8- and H5N1-infected mice at 3 and 7 dpi, although upregulation levels were more enhanced in H5N1-infected mice than in H5N8-infected mice. It is likely that the reason why inflammation pathways were found to be more elevated in H5N1-infected mice than in H5N8-infected mice was because there were higher numbers of statiscially significant DEGs found from H5N1-infected mice than those from H5N8-infected mice at 3 and 7 dpi, which is consistent with higher viral titres and virulence observed *in vivo*^7^.

**Figure 4:**
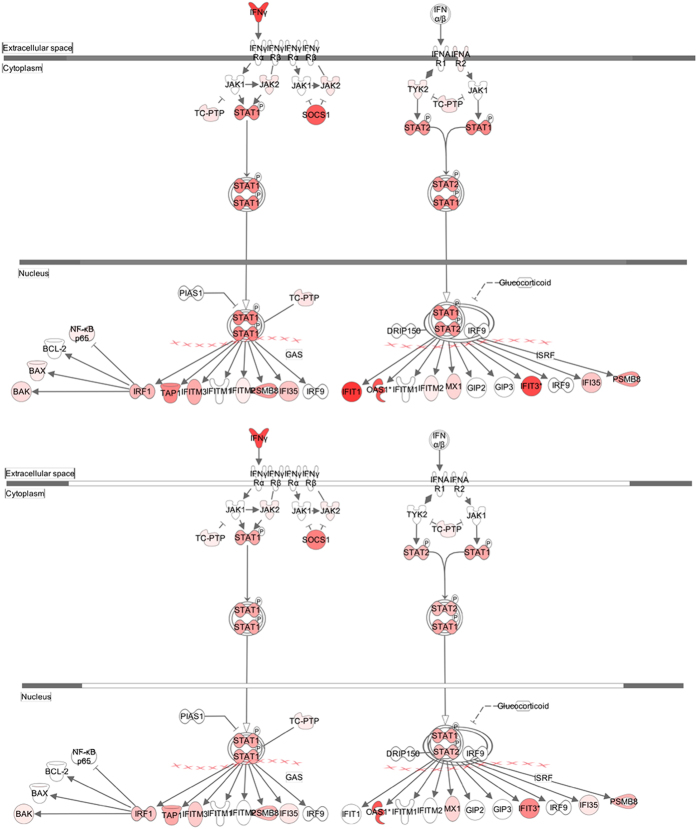
Pathway analysis for interferon signalling at 3 days post infection (dpi). Pathway analysis with IPA software allowed identification of pathways that were differentially expressed between H5N1- and H5N8- infected mice. Genes associated with the interferon signalling canonical pathway that showed differential expression are highlighted in colour. Colour intensity indicates the degree of upregulation (red) or downregulation (green) relative to the mock-infected mice. Solid lines represent direct interactions and dashed lines indirect interactions.

**Figure 5:**
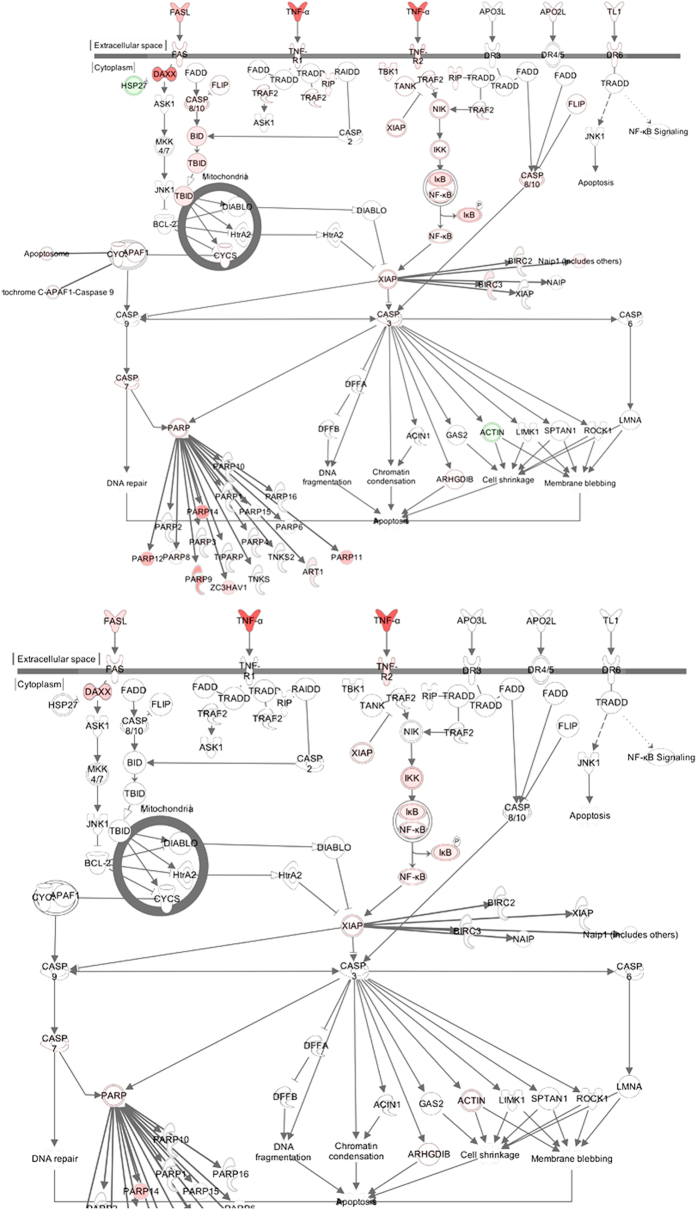
Pathway analysis for death receptor signalling at 3 days post infection (dpi). Pathway analysis with IPA software allowed identification of pathways that were differentially expressed between H5N1- and H5N8- infected mice. Genes associated with the death receptor signalling canonical pathway that showed differential expression are highlighted in colour. Colour intensity indicates the degree of upregulation (red) or downregulation (green) relative to the mock-infected mice. Solid lines represent direct interactions and dashed lines indirect interactions.

Next, the expression of different pathways was analysed with IPA, and several gene interaction networks were created. DEGs due to viral infection were indicated as either red (upregulated) or green (downregulated) (Figs 4 and 5, Supplementary Figs 5 and 6). We focused our analyses primarily on pathways and sets of genes with a previous association with interferon signalling at 3 dpi, as shown in Fig. 4. Notable differences included higher upregulation of key downstream signalling molecules such as Janus kinase/signal transducers and activators of transcription (JAK/STAT), interferon-induced protein with tetratricopeptide repeats (IFIT3), and 2′-5′-oligoadenylate synthetase 1 (OAS1) in H5N1-infected mice than in H5N8-infected mice. Similarly, a network map of pattern recognition receptor-recognizing viruses was created to illustrate receptor connections with many chemokines, chemokine receptors, and interferon-inducible genes for H5N8- and H5N1-infected mice at 3 dpi (Supplementary Fig. 5).

We further generated a network map of death receptor signalling for H5N8- and H5N1-infected mice at 3 dpi (Fig. 5). FasL, TNF-α, and Apo2L (TRAIL)-mediated downstream signalling leading to cell apoptosis were activated to a greater extent in H5N1-infected mice than in H5N8-infected mice. In addition, acute phase signalling was also activated more in H5N1-infected mice than in H5N8-infected mice at 7 dpi (Supplementary Fig. 6) (z-scores were 5.81 and 3.47 for H5N1 and H5N8, respectively), which also correlates with more severe pathogenicity observed in H5N1-infected mice. H5N1 viruses were very lethal and replicated systemically in various tissues in mice; A significant upregulation of death receptor signalling by H5N1 may have been responsible for the severe pathogenicity observed in the H5N1-infected mice *in vivo*. Consequently, these data collectively suggest that higher upregulation of DEGs from both viral and host transcriptomes induced by H5N1 infection at 3 and 7 dpi may have led to more robust immune activation, elevating persistent activation and recruitment of immune cells (e.g. T cells, macrophages, and monocytes) and resulting in cytokine storm-like severe pathogenicity in lethal HPAI A(H5N1) viral infection in mice.

## Discussion

Since 2013, cases of HPAI H5N8 infection in wild birds and poultry have emerged and caused economic and social losses globally, including in South Korea, China, Europe, and the United States^4^,^6^,^17^,^18^,^19^,^20^. In South Korea, HPAI A(H5N8) reassortment viruses emerged anew in domestic poultry, causing outbreaks in breeding ducks and chickens in mid-January 2014. Because the public health risk of the novel HPAI A(H5N8) virus to animals and humans remains uncertain, we aimed to evaluate underlying features of the pathogenicity of this virus in an animal model, using dual RNA-seq of host and virus.

Our data highlight distinct and dynamic changes in expression patterns of host genes following MDk/W452(H5N8) virus infection in mouse lungs compared with those observed following H5N1 virus infection. Previously, we have demonstrated that intranasal inoculation of mice with 10^6^ log_10_ EID_50_/mL (EID_50_ is 50 percent embryo infectious dose) of MDk/W452(H5N8) induced only 6% reduction in body weight and 40% lethality within the 14-day observation period, whereas EM/W149(H5N1) infection was highly lethal and replicated systemically in various tissues^7^. We observed a more modest secretion of inflammatory molecules in the organs of H5N8-infected mice than in those of H5N1-infected mice. Additionally, quantitative realtime PCR was performed to validate RNA-seq data and the transcript levels of cytokines/chemokines, such as CXCL10, CXCL11, IFN-γ, TNF-α and IL-24, were all up-regulated upon H5N1 and H5N8 infection (data not shown). These data correlate well with the RNA-seq data presented in this study, in which significantly upregulated DEGs were mostly associated with chemokine pathways, and H5N1-infected mice expressed higher levels of chemokines and interferon-stimulated genes (ISGs) than did H5N8-infected mice at 3 and 7 dpi (Fig. 3). A robust activation of immune system activation pathways such as acute phase response, p38 MAPK, and iNOS signalling in H5N1-infected mice correlates with high viral replication in these mice, and could play an important role in pathogenesis. Furthermore, our data demonstrating a more marked activation of the death receptor signalling pathway in H5N1-infected mice also correlates with the high pathogenicity observed with this virus *in vivo* (Fig. 5). Notably, when we analysed DEGs found only in the infected groups and not sequenced in the mock-infected group, few interesting novel genes and genes with unknown function in response to influenza virus infection were found. One of the most upregulated genes was IL-24, which is a new member of the IL-10 family of cytokines. Its role in virus-mediated immune responses was unknown. It may be worthwhile to carry out further studies to investigate the role of IL-24 in viral replication control.

Previously, our data have shown that virus titres from H5N8-infected mouse lungs were lower than those from H5N1-infected mouse lungs, suggesting a moderate viral replication pattern of H5N8 in comparison to that of H5N1^7^. Consistent with this, our RNA-seq analysis of virus genes also indicates that viral transcript counts for all eight genes (HA, M, NA, NP, NS, PA, PB1, and PB2) were lower in H5N8 than in H5N1 at 3 and 7 dpi. Interestingly, at 1 dpi, distinct patterns in viral transcript counts were observed in comparison to those observed at 3 and 7 dpi. The transcript counts for HA, NS1, and NA were significantly more elevated in H5N8-infected mouse lungs compared with H5N1-infected mouse lungs at 1 dpi. This raises the important question of whether H5N8’s ability to replicate with better efficiency at earlier post infection time points might have led to increased numbers of DEGs being involved in host anti-viral innate immune defence functions to suppress viral replication efficiently, thereby resulting in the consequent modest pathogenicity.

RNA-seq analysis of influenza virus infection has been performed in mouse, crow, and chicken samples^10^,^21^,^22^. In mice, pandemic H1N1/2009 virus infection activated strong virus-sensing signals, such as TLRs, NLRs, RIGs, as well as the NF-κB and JAK-STAT pathways, which play a significant role in inducing innate immunity^21^. Additionally, there are several transcriptome studies, suggesting the importance of understanding dynamics and magnitude of the innate immune responses in shaping the H5N1 pathogenesis^23^,^24^,^25^,^26^. These studies indicate more robust and early induction of inflammatory response observed in all animal models infected with H5N1 and up-regulated DEGs from H5N1-infected mice consist of genes involved in inflammation and apoptosis, which may have contributed to underlying mechanisms of severe pathogenicity. Similar to this, our data also indicate that avian influenza infection increased expression of interferon and innate immune virus-sensing pathways.

It was also interesting to note that IFN-γ was robustly expressed in the top 10 upregulated genes of H5N8-infected lungs. Previous studies demonstrate that IFN-γ treatment significantly reduced the number of T and NKT cells in the lungs at the inflammatory phase following infection, thus contributing to protection against viral pathogenesis^27^. In addition, according to Morrison *et al.*, H5N1-infected mice also induced an increased secretion of IFN-γ^24^. Based on our data, it is possible that at early time point (1 dpi), expression of immune-related DEGs from H5N8-infected mice may have facilitated an increase in IFN-γ production, which may have led to abrupt anti-viral immunity and thus limiting viral replication. On the other hand, a minimal induction of immune-related genes from H5N1–infected mice at early time point may have caused delayed IFN-γ expression, possibly contributing to the induction of the rapid replication of virus. Additionally, interferon γ-inducible protein (CXCL10), an important chemoattractant for T lymphocytes and monocytes, was more highly upregulated in the H5N1 group at 3 and 7 dpi, and this may have led to more robust immune activation, inducing persistent activation and recruitment of immune cells (e.g. T cells, macrophages, and monocytes). In line with this, our previous *in vivo* results suggest that the cytokine storm-like phenotype is the hallmark of severely lethal HPAI A(H5N1) virus infection in mice^7^.

Dual RNA-seq also revealed a good correlation between viral transcript levels and host gene expression levels. Accordingly, viral transcript counts of NS1 for H5N8 were also found to be higher than those for H5N1 at 1 dpi, suggesting a strong correlation between sequenced numbers of viral transcripts and expression levels of host immune-related genes in each virus-infected lung at 1 dpi. More H5N1 genes than H5N8 genes were sequenced at 3 and 7 dpi, which is consistent with the higher viral titres in infected lungs and the higher virulence observed *in vivo*. The increase in the replication of H5N1virus in lungs may have resulted in the induction of immune cell recruitment with robust production of chemokines (such as CXCL10 and CXCL11); in contrast, H5N8 replication was relatively moderate and thus caused a relatively moderate induction of these chemokines at both 3 and 7 dpi. This observation may reflect a positive correlation with host responses related to virus sensing and virus-mediated inflammation, such as TREM1 signalling, acute phase response signalling, and virus receptor signalling, as shown by the IPA heat map analysis (Fig. 3B). Collectively, the activated innate immune system might have directly caused more severe pathological injury to the lung tissue, as demonstrated by increased activation of death receptor signalling in H5N1-infected mice.

The present study is the first to characterise the dual host and virus transcriptome of H5N8-infected animals and compare it with that of recent H5N1 infection. Differential expression of specific factors observed between avian influenza virus strains could explain the variation in disease pathogenicity. These findings provide a framework for future studies examining the molecular mechanisms underlying the pathogenicity of H5N8. Taken together, our data provide a comprehensive transcriptome analysis of H5N8-infected mice, and several biological pathways related to anti-viral innate immunity and inflammation responses are involved in the highest regulated DEGs. Further global immunity studies on H5N8-vaccinated animals will provide valuable insight into understanding the immunogenicity of a novel H5N8 vaccine.

## Methods

### Ethics statement

All animal experiment protocols performed in this study followed general animal care guidelines mandated under the Guidelines for Animal Use and Care of the Korea Center for Disease Control (KCDC). They were approved by the Laboratory Animal Research Center (approval No. CBNUA-074-0904-01), which is a member of the Institutional Animal Care and Use Committee of Chungbuk National University, and were performed in a level 3 biosafety laboratory (BSL3) in Chungbuk National University (permit No. CBNU-BSL-14-001). The methods were carried out in accordance with the approved guidelines.

### Viruses

HPAI H5 viruses were isolated from wild bird faecal samples in the winters of 2006–2007 (A/environment/Korea/W149/2006 [EM/W149(H5N1)]), and 2013–2014 (A/mallard duck/Korea/W452/2014 [MDk/W452(H5N8)]) and grown in specific pathogen-free 10-day-old embryonated chicken eggs. Supernatants (allantoic fluids and cell culture) were harvested at 48 hours post inoculation (hpi) and aliquoted into cryovials (1 mL each). Stock viral titres were determined by EID_50_ and 50% tissue culture infectious dose (TCID_50_) end-point titrations. All experiments with HPAI H5 viruses were conducted in an enhanced biosafety level 3 (BSL3-1) containment facility approved by the KCDC.

### Virus infection in mice

Six-week-old female BALB/c mice (Samtako, Seoul, Korea) were used for all infection experiments. Ten mice per group were inoculated intranasally with 10^2^–10^7^ EID_50_/50 mL of MDk/W452(H5N8) and EM/W149(H5N1). Body weights and survival patterns were recorded daily for 14 days. For virological and pathological examinations, groups of mice were inoculated intranasally with 10^5^ EID_50_/50 mL of virus, and six mice per group were euthanised at 1, 3, 5, 7 and 9 dpi to examine the growth kinetics of the virus in mouse lungs.

### RNA quality check, library construction, and sequencing

RNA quality was assessed by analysis of rRNA band integrity on an Agilent RNA 6000 Nano Kit (Agilent Technologies, Santa Clara, CA). Prior to cDNA library construction, poly (A) mRNA was enriched using 2 μg of total RNA and magnetic beads with oligo(dT) primer. The purified mRNAs were then disrupted into short fragments, and double-stranded cDNAs were immediately synthesised. The cDNAs were subjected to end-repair, poly (A) addition, and connection with sequencing adapters using the TruSeq RNA Sample Prep Kit (Illumina, San Diego, CA). Suitable fragments, automatically purified using a BluePippin 2% agarose gel cassette (Sage Science, Beverly, MA), were selected as templates for polymerase chain reaction (PCR) amplification. The final library sizes and qualities were evaluated electrophoretically using an Agilent High Sensitivity DNA Kit (Agilent Technologies, Santa Clara, CA), and the fragment was found to be between 350 and 450 bp. Subsequently, the library was sequenced using an Illumina HiSeq 2500 sequencer (Illumina, San Diego, CA). Three biological replicates from RNA-seq run were performed per time point. RNA-seq data from this study have been submitted to the NCBI Sequence Read Archive (SRA) under the accession number of PRJNA290088(SUB1025618) (http://www.ncbi.nlm.nih.gov/sra/).

### Transcriptome data analysis

Low-quality reads were filtered according to the following criteria: (i) reads containing more than 10% of skipped bases (marked as ‘N’s); (ii) reads containing more than 40% of bases with quality scores less than 20; and (iii) reads with average quality scores of each read of less than 20. The whole filtering process was performed using in-house scripts. Filtered reads were mapped to the mouse reference genome (Ensembl release 77)^28^ using the aligner STAR v.2.3.0e^29^. Gene expression level was measured with Cufflinks v2.1.1^30^ using the gene annotation database of Ensembl release 77. Non-coding gene regions were removed with the mask option. To improve the accuracy of measurement, multi-read-correction and frag bias-correct options were applied. All other options were set to default values. For differential expression analysis, gene-level count data were generated using the HTSeq-count v0.5.4p3^31^ tool with the options “-m intersection-nonempty” and “–r option considering paired-end sequence.” Based on the calculated read count data, DEGs were identified using the R package called TCC^32^. The TCC package applies robust normalisation strategies to compare tag count data. Normalisation factors were calculated using the iterative DEGES/edgeR method. The *q*-value was calculated based on the *p*-value using the *p* adjust function of the R package with default parameter settings. DEGs were identified based on a false discovery rate (FDR) *q*-value threshold of less than 0.05. Genes were considered differentially expressed with displaying a change of more than two-fold with a *q*-value of less than or equal to 0.05 for at least one time point.

### Gene ontology (GO) enrichment analysis

The GO database classifies genes according to the three categories of biological process, cellular component, and molecular function, and predicts the function of the selected genes. To characterise the identified genes from DEG analysis, a GO-based trend test was performed using Fisher’s exact test; *p*-values < 0.001 were considered statistically significant.

### Ingenuity Pathway Analysis (IPA) analysis

Data were analysed using QIAGEN’s IPA (QIAGEN, Redwood City, USA). The canonical pathways and functional processes of biological importance that were the most significant were identified using the list of DEGs identified with RNA-seq and the Ingenuity Pathways Knowledge Base. The Ingenuity Knowledge Base contains the largest database of manually curated and experimentally validated physical, transcriptional, and enzymatic molecular interactions. Furthermore, each interaction in the Ingenuity Knowledge Base is supported by previously published information. The list of DEGs was overlaid onto a global molecular network developed from information contained in the Ingenuity Pathways Knowledge Base, and networks were then algorithmically generated on the basis of connectivity. Pathway enrichment *p*-values (Fisher’s exact test) and activation z-scores were calculated by IPA. mRNAs that were upregulated or downregulated at least 2-fold at each time point were included for IPA analysis.

## Additional Information

**How to cite this article**: Park, S.-J. *et al.* Dynamic changes in host gene expression associated with H5N8 avian influenza virus infection in mice. *Sci. Rep.* **5**, 16512; doi: 10.1038/srep16512 (2015).

## Supplementary Material

Supplementary Information

## Figures and Tables

**Table 1:** Top 10 up-regulated differentially expressed genes (DEGs) in H5N8- and H5N1-infected mice.

Dpi	H5N8	Log2FC	p-value	H5N1	Log2FC	p-value
1 dpi	Keratin 1	6.56	0.00005	RIKEN cDNA C920009B18 gene	7.43	0.00005
	Prostate stem cell antigen	6.37	0.0001	Moloney leukemia virus 10-like 1	3.05	0.00005
	Gastric intrinsic factor	6.07	0.00005	Nuclear receptor subfamily 1, group D, member 1	2.55	0.00005
	Chemokine (C-X-C motif) ligand 3	6.07	0.0001	Unknown	2.47	0.00005
	Serum amyloid A 3	5.42	0.00005	Prostaglandin D2 synthase (brain)	2.46	0.00005
	Mucin 5, subtypes A and C, tracheobronchial/gastric	4.55	0.00005	Myostatin	2.29	0.00005
	Keratin 10	4.44	0.00005	Keratin 6B	2.26	0.00005
	Secreted and transmembrane 1B	4.36	0.00005	Envelope glycoprotein	2.19	0.00005
	Keratin 6B	3.92	0.00005	Protease, serine 30	2.17	0.0003
	UDP glucuronosyltransferase 2 family, polypeptide B34	3.81	0.0006	Myosin, light polypeptide 2, regulatory, cardiac, slow	2.17	0.00005
3 dpi	Immunoresponsive gene 1	9.1	0.00005	Chemokine (C-X-C motif) ligand 10	8.84	0.00005
	Chemokine (C-X-C motif) ligand 10	7.83	0.00205	Gag-Pol polyprotein	8.72	0.00005
	Gag-Pol polyprotein	7.58	0.00005	Immunoresponsive gene 1	8.08	0.00005
	Serum amyloid A 3	7.53	0.0002	Chemokine (C-C motif) ligand 7	7.6	0.00005
	Chemokine (C-X-C motif) ligand 9	7.02	0.00005	Serum amyloid A 3	7.56	0.00005
	Indoleamine 2,3-dioxygenase 1	6.96	0.00005	RIKEN cDNA C920009B18 gene	7.48	0.00005
	Lymphocyte antigen 6 complex, locus I	6.17	0.00005	Chemokine (C-C motif) ligand 2	7.12	0.00005
	Interferon gamma	6.14	0.00005	Unknown	7.06	0.0045
	Chemokine (C-C motif) ligand 7	6.14	0.00005	Myxovirus (influenza virus) resistance 1	6.9	0.0045
	Chemokine (C-X-C motif) ligand 2	6.13	0.00005	Tripartite motif-containing 30D	6.79	0.00005
7 dpi	Ig heavy chain V region MC101	10.2	0.00805	Chemokine (C-X-C motif) ligand 11	10.4	0.021
	RIKEN cDNA C920009B18 gene	8.83	0.0009	Gag-Pol polyprotein	9.78	0.00005
	chemokine (C-X-C motif) ligand 11	8.76	0.01225	Immunoresponsive gene 1	9.21	0.00005
	Gag-Pol polyprotein	8.03	0.00005	Chemokine (C-C motif) ligand 7	9.08	0.00005
	Indoleamine 2,3-dioxygenase 1	7.43	0.00005	Chemokine (C-X-C motif) ligand 10	8.99	0.00005
	Chemokine (C-X-C motif) ligand 10	7.36	0.00005	Serum amyloid A 3	8.72	0.00005
	Chemokine (C-C motif) ligand 7	7.22	0.00005	RIKEN cDNA C920009B18 gene	8.64	0.00005
	Granzyme B	6.75	0.00005	Chemokine (C-C motif) ligand 2	8.52	0.00005
	Immunoresponsive gene 1	6.73	0.00005	Chemokine (C-X-C motif) ligand 9	8.22	0.00005
	Interferon gamma	6.56	0.00005	Granzyme B	7.86	0.00005

**Table 2:** Top 10 down-regulated differentially expressed genes (DEGs) in H5N8- and H5N1-infected mice.

Dpi	H5N8	Log_2_ FC	p-value	H5N1	Log_2_ FC	p-value
1 dpi	Myosin, light polypeptide 2, regulatory, cardiac, slow	−8.3	0.00005	Heat shock 70 kDa protein 1A	−3.35	0.00005
	Immunoglobulin heavy variable 1-73	−7.25	0.00005	Unknown	−3.21	0.00115
	Natriuretic peptide type A	−4.48	0.00005	Phosphoenolpyruvate carboxykinase 1, cytosolic	−2.9	0.00005
	Heat shock 70 kDa protein 1A	−3.83	0.00005	Neutrophilic granule protein	−2.71	0.00005
	Myosin binding protein C, cardiac	−3.23	0.00005	Chemokine (C-X-C motif) ligand 5	−2.39	0.00075
	Unknown	−3.19	0.00005	Unknown	−2.35	0.00005
	Chloride channel calcium activated 3	−3.05	0.00005	Neuronal PAS domain protein 2	−2.34	0.00005
	LINE-1 reverse transcriptase homolog	−3.01	0.0031	Monoacylglycerol O-acyltransferase 1	−2.21	0.0014
	Corin	−2.9	0.00005	Arachidonate 15-lipoxygenase	−2.16	0.00005
	Phosphoenolpyruvate carboxykinase 1, cytosolic	−2.79	0.00005	Ankyrin repeat and EF−hand domain containing 1	−2.04	0.00005
	Immunoglobulin heavy variable 1-73	−3.97	0.00005	ATPase, H+ transporting, lysosomal V1 subunit B1	−4.55	0.00005
	Unknown	−3.96	0.00085	Unknown	−4.25	0.0026
	Thyroid stimulating hormone receptor	−2.74	0.00025	Immunoglobulin heavy variable 1-73	−4.08	0.00005
	Family with sequence similarity 124, member B	−2.5	0.00005	Cytochrome P450 4A12A	−3.51	0.00005
	Neuronal PAS domain protein 2	−2.34	0.00005	Cadherin-like 26	−3.43	0.00005
3 dpi	A disintegrin-like and metallopeptidase	−2.3	0.00005	Ig kappa chain V-IV region S107B	−3.42	0.0055
	Heat shock 70 kDa protein 1A	−2.28	0.00005	Cellular retinoic acid binding protein I	−3.15	0.0081
	Solute carrier family 17 (sodium phosphate), member 2	−2.17	0.00075	CD209a antigen	−3.09	0.00005
	phosphoenolpyruvate carboxykinase 1, cytosolic	−2.14	0.00005	Unknown	−3.06	0.00005
	Ring finger protein 112	−2.05	0.0018	Neuronal PAS domain protein 2	−3.03	0.00005
	Myosin, light polypeptide 2, regulatory, cardiac, slow	−9.79	0.00005	Hemoglobin alpha, adult chain 2	−7.44	0.00005
7 dpi	Bone morphogenetic protein 10	−5.08	0.00005	Hemoglobin, beta adult t chain	−7.25	0.00005
	Unknown	−3.95	0.0001	Hemoglobin alpha, adult chain 1	−7.01	0.00005
	CD209a antigen	−3.67	0.00005	Aminolevulinic, beta adult s chain	−6.75	0.00005
	Unknown	−3.55	0.00005	Haemoglobin, beta adult s chain	−6.7	0.00005
	Unknown	−3.38	0.00005	Unknown	−6.39	0.00005
	Cytochrome P450, family 1, subfamily a, polypeptide 1	−3.27	0.00005	Fatty acid binding protein 1, liver	−5.6	0.00265
	Unknown	−3.23	0.00395	Lactase-like	−5.58	0.0198
	Cadherin-like 26	−3.12	0.00005	Unknown	−5.12	0.00005
	Immunoglobulin heavy variable 1-73	−3.1	0.00005	Solute carrier family 17 (sodium phosphate), number 2	−5.08	0.00005

## References

[b1] Alexander D. J., Parsons G. & Manvell R. J. Experimental assessment of the pathogenicity of eight avian influenza A viruses of H5 subtype for chickens, turkeys, ducks and quail. Avian pathology : journal of the WVPA 15, 647–662 (1986). 10.1080/03079458608436328 18766567

[b2] Zhao K. *et al.* Characterization of three H5N5 and one H5N8 highly pathogenic avian influenza viruses in China. Veterinary microbiology 163, 351–357 (2013). 23375651 10.1016/j.vetmic.2012.12.025

[b3] Fan S. *et al.* A novel highly pathogenic H5N8 avian influenza virus isolated from a wild duck in China. Influenza and other respiratory viruses 8, 646–653 (2014). 25363159 10.1111/irv.12289 PMC4262280

[b4] Wu H. *et al.* Novel reassortant influenza A(H5N8) viruses in domestic ducks, eastern China. Emerging infectious diseases 20, 1315–1318 (2014). 25075453 10.3201/eid2008.140339 PMC4111196

[b5] Lee Y. J. *et al.* Novel reassortant influenza A(H5N8) viruses, South Korea, 2014. Emerging infectious diseases 20, 1087–1089 (2014). 24856098 10.3201/eid2006.140233 PMC4036756

[b6] Kang H. M. *et al.* Novel Reassortant Influenza A(H5N8) Viruses among Inoculated Domestic and Wild Ducks, South Korea, 2014. Emerging infectious diseases 21, 298–304 (2015). 25625281 10.3201/eid2102.141268 PMC4313655

[b7] Kim Y.-I. *et al.* Pathobiological features of a novel, highly pathogenic avian influenza A(H5N8) virus. Emerging Microbes & Infections 3, e75 (2014). 26038499 10.1038/emi.2014.75 PMC4217095

[b8] Gavazzi G. & Krause K. H. Ageing and infection. The Lancet Infectious diseases 2, 659–666 (2002). 12409046 10.1016/s1473-3099(02)00437-1

[b9] Mei B., Ding X., Xu H. Z. & Wang M. T. Global gene expression changes in human peripheral blood after H7N9 infection. Gene 551, 255–260 (2014). 25192803 10.1016/j.gene.2014.08.062

[b10] Wang Y., Lupiani B., Reddy S. M., Lamont S. J. & Zhou H. RNA-seq analysis revealed novel genes and signaling pathway associated with disease resistance to avian influenza virus infection in chickens. Poultry science 93, 485–493 (2014). 10.3382/ps.2013-03557 24570473

[b11] Ertl R. & Klein D. Transcriptional profiling of the host cell response to feline immunodeficiency virus infection. Virology journal 11, 52 (2014). 24642186 10.1186/1743-422X-11-52 PMC3999937

[b12] Jones M. *et al.* RNA-seq analysis of host and viral gene expression highlights interaction between varicella zoster virus and keratinocyte differentiation. PLoS pathogens 10, e1003896 (2014). 24497829 10.1371/journal.ppat.1003896 PMC3907375

[b13] Rossetto C. C., Tarrant-Elorza M., Verma S., Purushothaman P. & Pari G. S. Regulation of viral and cellular gene expression by Kaposi’s sarcoma-associated herpesvirus polyadenylated nuclear RNA. Journal of virology 87, 5540–5553 (2013). 23468496 10.1128/JVI.03111-12 PMC3648157

[b14] Juranic Lisnic V. *et al.* Dual analysis of the murine cytomegalovirus and host cell transcriptomes reveal new aspects of the virus-host cell interface. PLoS pathogens 9, e1003611 (2013). 24086132 10.1371/journal.ppat.1003611 PMC3784481

[b15] Gupta P. *et al.* mda-7/IL-24: multifunctional cancer-specific apoptosis-inducing cytokine. Pharmacology & therapeutics 111, 596–628 (2006). 16464504 10.1016/j.pharmthera.2005.11.005 PMC1781515

[b16] Tessarz A. S. & Cerwenka A. The TREM-1/DAP12 pathway. Immunology letters 116, 111–116 (2008). 18192027 10.1016/j.imlet.2007.11.021

[b17] Avian influenza: no clear indication of how H5N8 virus entered the EU. The Veterinary record 176, 59 (2015). 10.1136/vr.h198 25598458

[b18] Jhung M. A. & Nelson D. I. Outbreaks of Avian Influenza A (H5N2), (H5N8), and (H5N1) Among Birds - United States, December 2014-January 2015. MMWR Morbidity and mortality weekly report 64, 111 (2015). 25654614 PMC4584850

[b19] Ip H. S. *et al.* Novel eurasian highly pathogenic avian influenza a h5 viruses in wild birds, washington, USA, 2014. Emerging infectious diseases 21, 886–890 (2015). 25898265 10.3201/eid2105.142020 PMC4412248

[b20] Harder T. *et al.* Influenza A(H5N8) Virus Similar to Strain in Korea Causing Highly Pathogenic Avian Influenza in Germany. Emerging infectious diseases 21, 860–863 (2015). 25897703 10.3201/eid2105.141897 PMC4414090

[b21] Zou W. *et al.* Insights into the increasing virulence of the swine-origin pandemic H1N1/2009 influenza virus. Scientific reports 3, 1601 (2013). 23549303 10.1038/srep01601 PMC3615340

[b22] Vijayakumar P. *et al.* Analysis of the crow lung transcriptome in response to infection with highly pathogenic H5N1 avian influenza virus. Gene 559, 77–85 (2015). 25592823 10.1016/j.gene.2015.01.016

[b23] Josset L., Tisoncik-Go J. & Katze M. G. Moving H5N1 studies into the era of systems biology. Virus research 178, 151–167 (2013). 23499671 10.1016/j.virusres.2013.02.011 PMC3834220

[b24] Morrison J. *et al.* H7N9 and other pathogenic avian influenza viruses elicit a three-pronged transcriptomic signature that is reminiscent of 1918 influenza virus and is associated with lethal outcome in mice. Journal of virology 88, 10556–10568 (2014). 24991006 10.1128/JVI.00570-14 PMC4178843

[b25] Tchitchek N. *et al.* Specific mutations in H5N1 mainly impact the magnitude and velocity of the host response in mice. BMC systems biology 7, 69 (2013). 23895213 10.1186/1752-0509-7-69 PMC3750405

[b26] Fornek J. L. *et al.* A single-amino-acid substitution in a polymerase protein of an H5N1 influenza virus is associated with systemic infection and impaired T-cell activation in mice. Journal of virology 83, 11102–11115 (2009). 19692471 10.1128/JVI.00994-09 PMC2772766

[b27] Weiss I. D. *et al.* IFN-gamma treatment at early stages of influenza virus infection protects mice from death in a NK cell-dependent manner. Journal of interferon & cytokine research: the official journal of the International Society for Interferon and Cytokine Research 30, 439–449 (2010). 10.1089/jir.2009.0084 20235626

[b28] Flicek P. *et al.* Ensembl 2013. Nucleic acids research 41, D48–55 (2013). 23203987 10.1093/nar/gks1236 PMC3531136

[b29] Dobin A. *et al.* STAR: ultrafast universal RNA-seq aligner. Bioinformatics 29, 15–21 (2013). 23104886 10.1093/bioinformatics/bts635 PMC3530905

[b30] Trapnell C. *et al.* Transcript assembly and quantification by RNA-Seq reveals unannotated transcripts and isoform switching during cell differentiation. Nature biotechnology 28, 511–515 (2010). 10.1038/nbt.1621 PMC3146043 20436464

[b31] Anders S., Pyl P. T. & Huber W. HTSeq–a Python framework to work with high-throughput sequencing data. Bioinformatics 31, 166–169 (2015). 25260700 10.1093/bioinformatics/btu638 PMC4287950

[b32] Sun J., Nishiyama T., Shimizu K. & Kadota K. TCC: an R package for comparing tag count data with robust normalization strategies. BMC bioinformatics 14, 219 (2013). 23837715 10.1186/1471-2105-14-219 PMC3716788

