# Erratum: Dynamic changes in host gene expression associated with H5N8 avian influenza virus infection in mice

**DOI:** 10.1038/srep20269

**Published:** 2016-01-29

**Authors:** Su-Jin Park, Mukesh Kumar, Hyeok-il Kwon, Rak-Kyun Seong, Kyudong Han, Jae-min Song, Chul-Joong Kim, Young-Ki Choi, Ok Sarah Shin

Scientific Reports 5: Article number: 1651210.1038/srep16512; published online: 11182015; updated: 01292016

This Article contains errors.

In Table 2, the number of genes associated with 1, 3 and 7 dpi were incorrectly presented. The correct Table 2 appears below as Table 1.

In addition, the Figure labels were omitted in Figures 4 and 5. The correct Figures 4 and 5 appear below as Figs 1 and 2 respectively.

And lastly, the Acknowledgements section is incomplete.

“This study was supported by a grant of the TEPIK (Transgovernmental Enterprise for Pandemic Influenza in Korea), which part of Korea Healthcare technology R&D Project by Ministry of Health & Welfare, Republic of Korea.(Grant No. A103001).”

should read:

“This study was supported by a grant of the TEPIK (Transgovernmental Enterprise for Pandemic Influenza in Korea), which part of Korea Healthcare technology R&D Project by Ministry of Health & Welfare, Republic of Korea.(Grant No. A103001) and grant number P30GM114737, from the Centers of Biomedical Research Excellence program of the National Institute of General Medical Sciences, National Institutes of Health.”

## Figures and Tables

**Figure 1:**
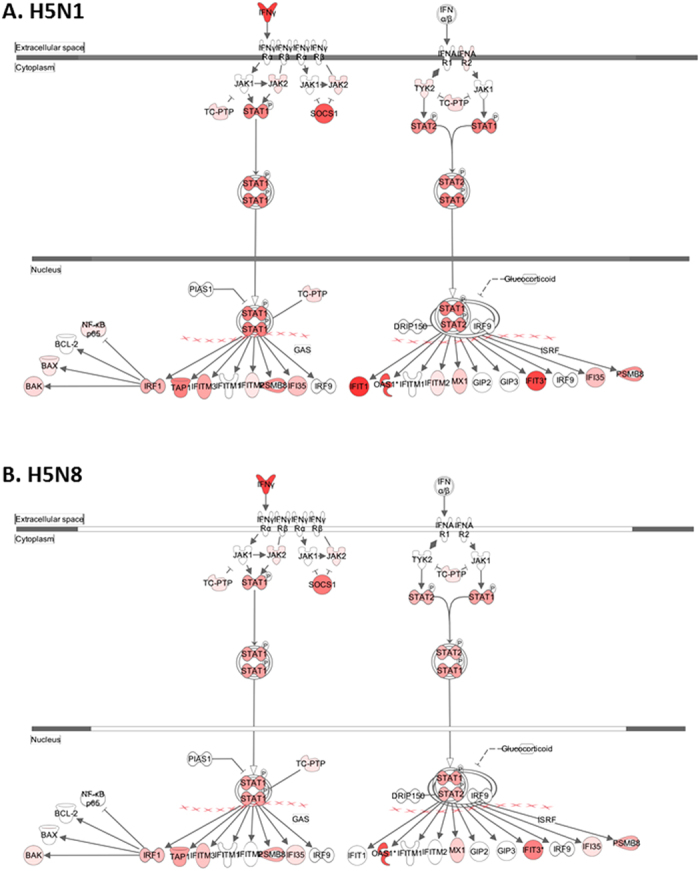


**Figure 2:**
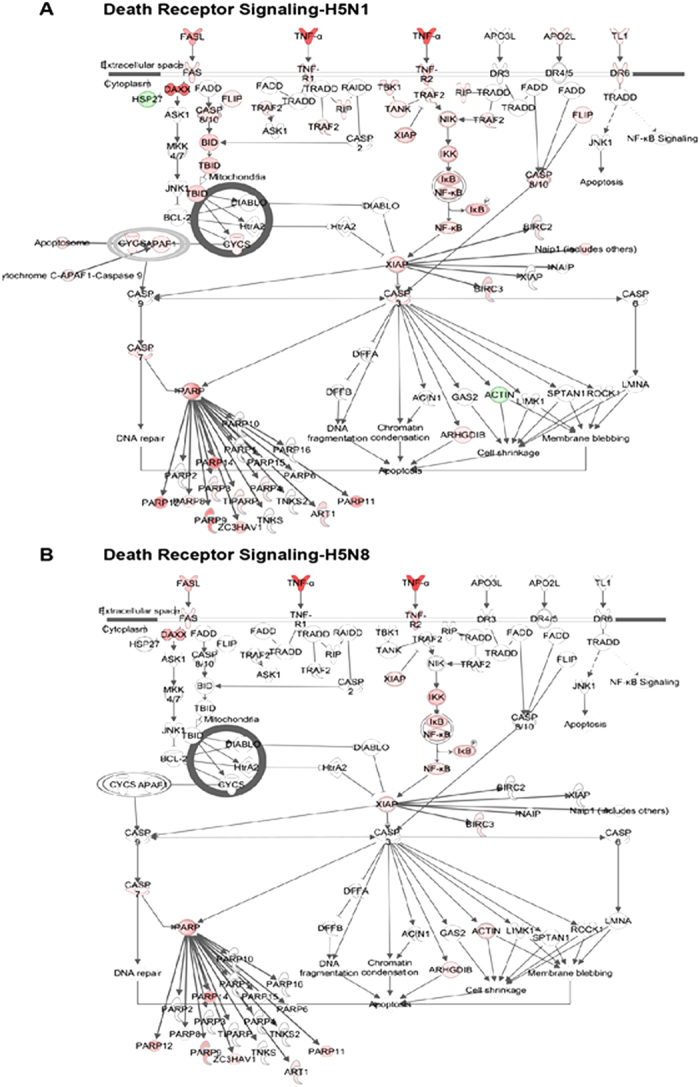


**Table 1:** 

**Dpi**	**H5N8**	**Log**_**2**_ **FC**	**p-value**	**H5N1**	**Log**_**2**_ **FC**	**p-value**
1 dpi	Myosin, light polypeptide 2, regulatory, cardiac, slow	−8.3	0.00005	Heat shock 70 kDa protein 1A	−3.35	0.00005
	Immunoglobulin heavy variable 1-73	−7.25	0.00005	Unknown	−3.21	0.00115
	Natriuretic peptide type A	−4.48	0.00005	Phosphoenolpyruvate carboxykinase 1, cytosolic	−2.9	0.00005
	Heat shock 70 kDa protein 1A	−3.83	0.00005	Neutrophilic granule protein	−2.71	0.00005
	Myosin binding protein C, cardiac	−3.23	0.00005	Chemokine (C-X-C motif) ligand 5	−2.39	0.00075
	Unknown	−3.19	0.00005	Unknown	−2.35	0.00005
	Chloride channel calcium activated 3	−3.05	0.00005	Neuronal PAS domain protein 2	−2.34	0.00005
	LINE-1 reverse transcriptase homolog	−3.01	0.0031	Monoacylglycerol O-acyltransferase 1	−2.21	0.0014
	Corin	−2.9	0.00005	Arachidonate 15-lipoxygenase	−2.16	0.00005
	Phosphoenolpyruvate carboxykinase 1, cytosolic	−2.79	0.00005	Ankyrin repeat and EF-hand domain containing 1	−2.04	0.00005
3 dpi	Immunoglobulin heavy variable 1-73	−3.97	0.00005	ATPase, H+ transporting, lysosomal V1 subunit B1	−4.55	0.00005
	Unknown	−3.96	0.00085	Unknown	−4.25	0.0026
	Thyroid stimulating hormone receptor	−2.74	0.00025	Immunoglobulin heavy variable 1-73	−4.08	0.00005
	Family with sequence similarity 124, member B	−2.5	0.00005	Cytochrome P450 4A12A	−3.51	0.00005
	Neuronal PAS domain protein 2	−2.34	0.00005	Cadherin-like 26	−3.43	0.00005
	A disintegrin-like and metallopeptidase	−2.3	0.00005	Ig kappa chain V-IV region S107B	−3.42	0.0055
	Heat shock 70 kDa protein 1A	−2.28	0.00005	Cellular retinoic acid binding protein I	−3.15	0.0081
	Solute carrier family 17 (sodium phosphate), member 2	−2.17	0.00075	CD209a antigen	−3.09	0.00005
	phosphoenolpyruvate carboxykinase 1, cytosolic	−2.14	0.00005	Unknown	−3.06	0.00005
	Ring finger protein 112	−2.05	0.0018	Neuronal PAS domain protein 2	−3.03	0.00005
7 dpi	Myosin, light polypeptide 2, regulatory, cardiac, slow	−9.79	0.00005	Hemoglobin alpha, adult chanin 2	−7.44	0.00005
	Bone morphogenetic protein 10	−5.08	0.00005	Hemoglobin, beta adult t chain	−7.25	0.00005
	Unknown	−3.95	0.0001	Hemoglobin alpha, adult chain 1	−7.01	0.00005
	CD209a antigen	−3.67	0.00005	Aminolevulinic, beta adult s chain	−6.75	0.00005
	Unknown	−3.55	0.00005	Haemoglobin, beta adult s chain	−6.7	0.00005
	Unknown	−3.38	0.00005	Unknown	−6.39	0.00005
	Cytochrome P450, family 1, subfamily a, polypeptide 1	−3.27	0.00005	Fatty acid binding protein 1, liver	−5.6	0.00265
	Unknown	−3.23	0.00395	Lactase-like	−5.58	0.0198
	Cadherin-like 26	−3.12	0.00005	Unknown	−5.12	0.00005
	Immunoglobulin heavy variable 1-73	−3.1	0.00005	Solute carrier family 17 (sodium phosphate), number 2	−5.08	0.00005

