# Supplementary material for: Dynamic changes in host gene expression associated with H5N8 avian influenza virus infection in mice
Source: Sci Rep. 2015 Nov 18;5:16512. doi: 10.1038/srep16512 (PMC4649622; doi:10.1038/srep16512)
Supplement: Supplementary Information [file srep16512-s1.doc]

**Dynamic changes in host gene expression associated with H5N8 avian influenza virus infection in mice**

Authors:

Su-Jin Park^1^, Mukesh Kumar^2^, Hyeok-il Kwon^1^, Rak-Kyun Seong^3^, Kyudong Han^4^, Jae-min Song^5^, Chul-Joong Kim^6^, Young-Ki Choi^1^* and Ok Sarah Shin^3,7^*

Author affiliations:

^1^College of Medicine and Medical Research Institute, Chungbuk National University, Cheongju 361-763, Republic of Korea

^2^Department of Tropical Medicine, Medical Microbiology and Pharmacology, Pacific Center for Emerging Infectious Diseases Research, John A. Burns School of Medicine, University of Hawaii at Manoa, Honolulu, HI, 96822, USA

^3^Department of Biomedical Sciences, College of Medicine, Korea University Guro Hospital, Seoul, 152-703, Republic of Korea

^4^Department of Nanobiomedical Science, Dankook University, Cheonan, 330-714 Republic of Korea

^5^Department of Global Medical Science, Sungshin Women's University, Seoul, 136-742 Republic of Korea

^6^College of Veterinary Medicine, Chungnam National University, Daejeon, 305-764 Republic of Korea

^7^Department of Microbiology, College of Medicine, Korea University, Seoul, 136-701 Republic of Korea

*YKC and OSS contributed equally to this work

Correspondence and requests for materials should be addressed to Y.K.C and O.S.S (phone: 82226263280, fax 82226261962, email; [choiki55@chungbuk.ac.kr](mailto:choiki55@chungbuk.ac.kr) and [oshin@korea.ac.kr](mailto:oshin@korea.ac.kr))

**Supplementary Figures**

**Supplementary Fig. 1. Sequencing quality check**

(a) Sequencing quality check. (b) Gene coverage of each sample is shown. The difference in colour represents the percent of bases with corresponding depth in the whole gene under the corresponding volume of sequencing bases. Pie charts indicate the proportion of RNA-seq reads assigned to assembled transcripts, previous gene annotations, or unassignable reads occurring in intronic or intergenic regions. (c) Alignment statistics showing the number of reads corresponding to uniquely mapped, mapped, unmapped, and low quality reads. Sequence reads matches against reference genomes. Matches of sequence reads in BLAST alignment against sequenced reference genomes.

**
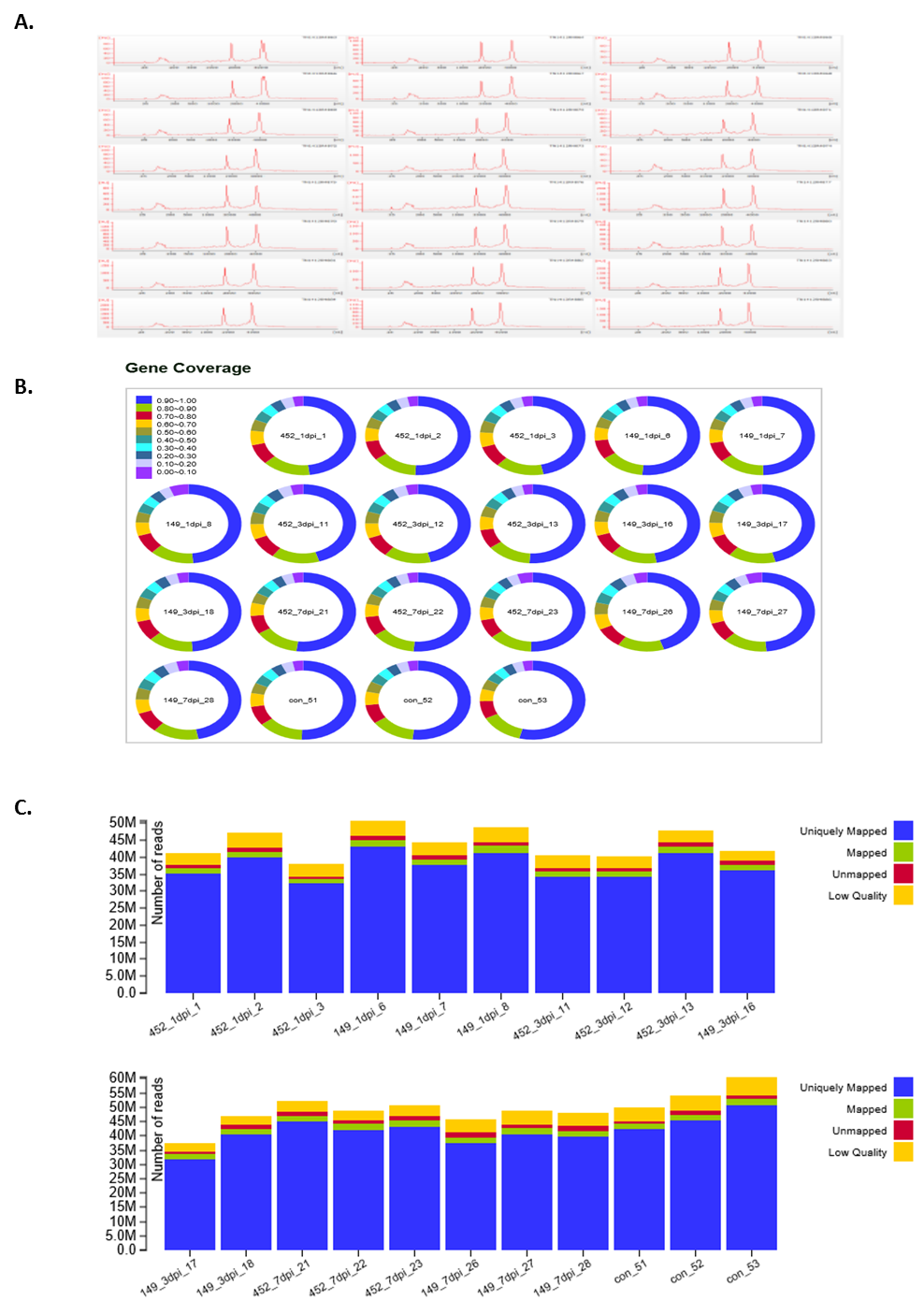
**

**Supplementary Fig. 2. Hierarchical clustering, scatter plot, and MA plot**

Hierarchical clustering heatmap of H5N8- *vs* H5N1-infected mice (a). The heat map shows similarities of biological replicates within a group. Red, black, and blue indicate gene expression above, equal to, and below the mean, respectively. (b) Scatter plot. The x-axis shows expression levels of genes from mock-infected groups, whereas the y-axis shows expression levels of genes from virus-infected groups. Red dots are relatively highly expressed genes in virus-infected groups, whereas blue dots represent relatively highly expressed genes in mock-infected groups. (c) MA plot. The M (log fold change) of each transcript between mock and infected pairs is plotted against A (average log expression level) of each mock and infected pair. In these plots, each point represents an annotated transcript. The black dots reflect no change and the red dots represent transcripts with 2-fold change by edgeR analysis.

**
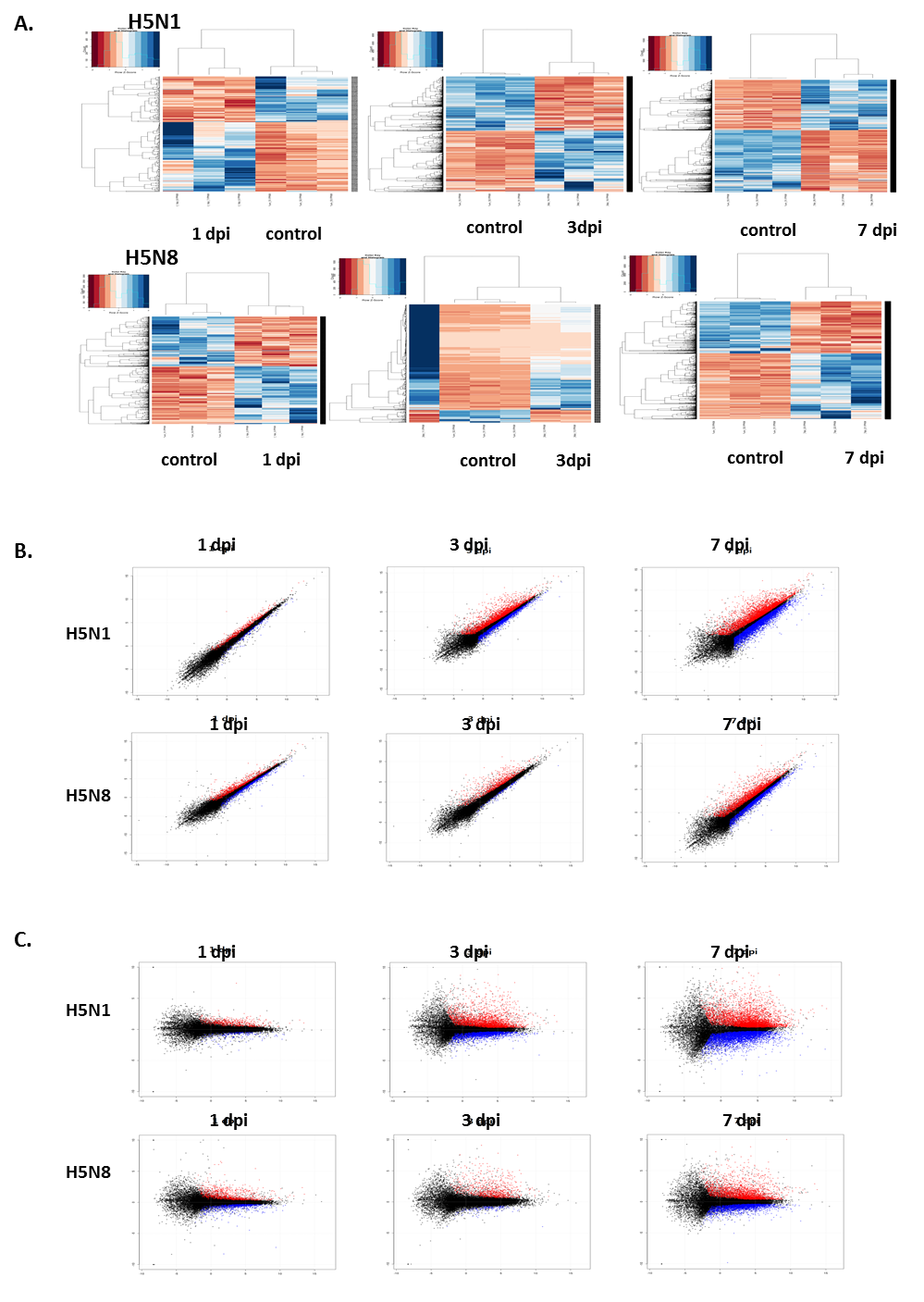
**

**Supplementary Fig. 3. Three-dimensional multi-dimensional scaling (3D-MDS) plots** 3D-MDS were generated based on all pairwise distances between the global transcriptome-wide RNA-seq profiles of the samples shown here. H5N1 and H5N8 infected samples at 3 and 7 dpi show an obvious wide distribution than mock control and H5N1- or H5N8-infected samples at 1 dpi.


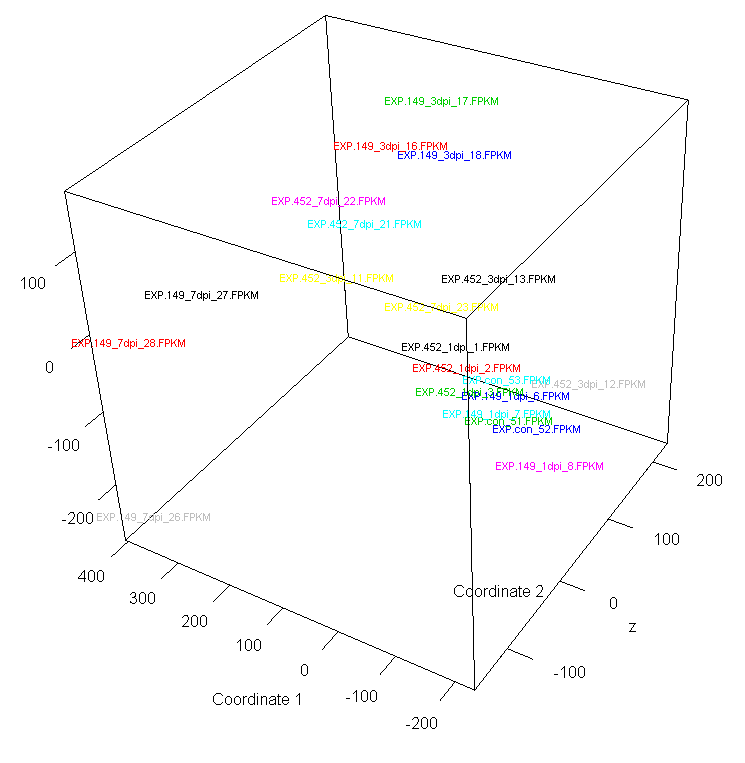


**Supplementary Fig. 4. Gene ontology (GO) Enrichment analysis (biological process)**

Number of upregulated genes for H5N1 and H5N8-infected sample at 1, 3, and 7 dpi are shown.


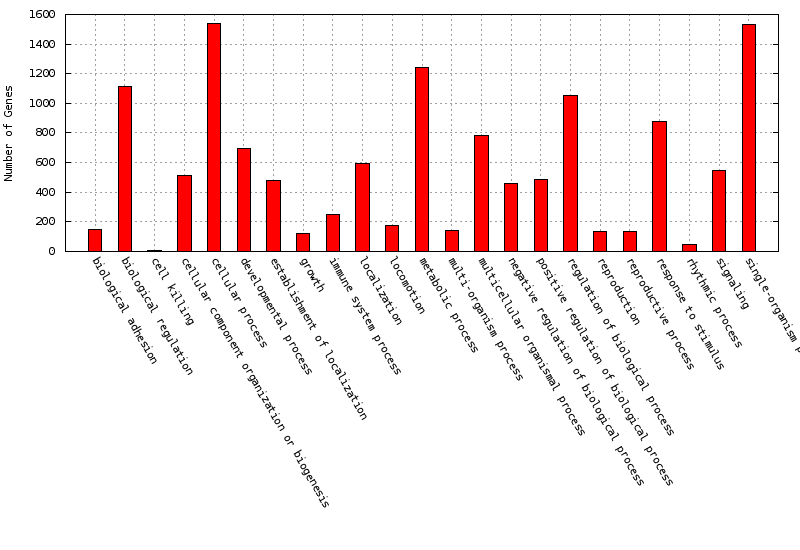

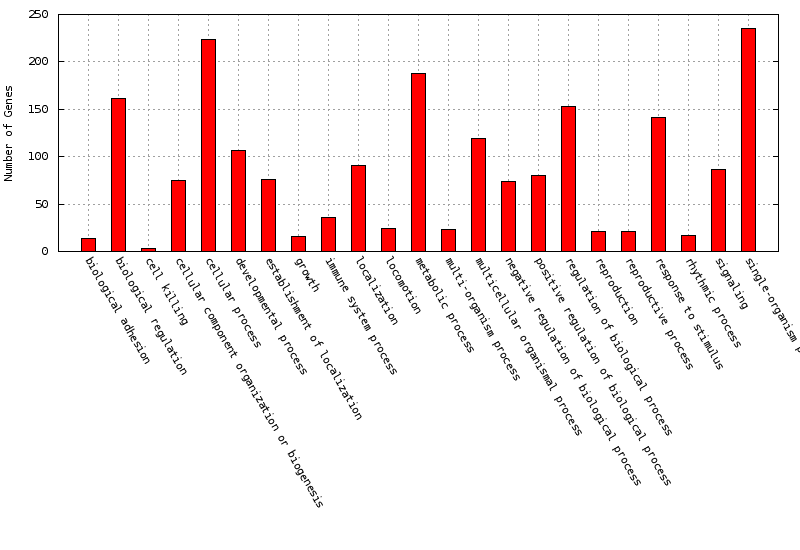


H5N8 1dpi

H5N1 1dpi


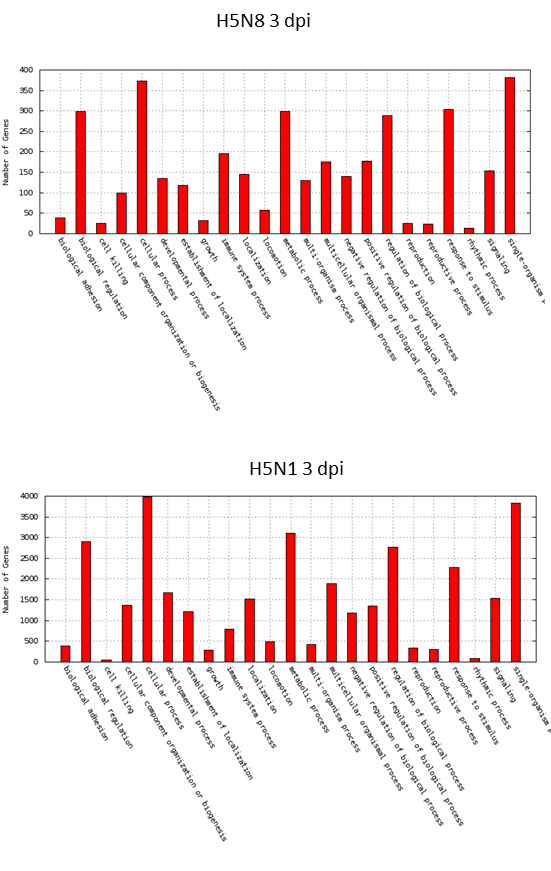


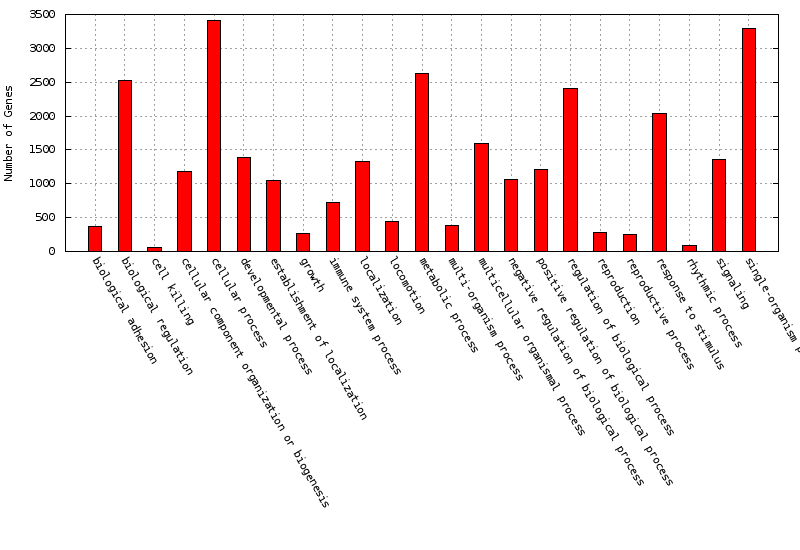

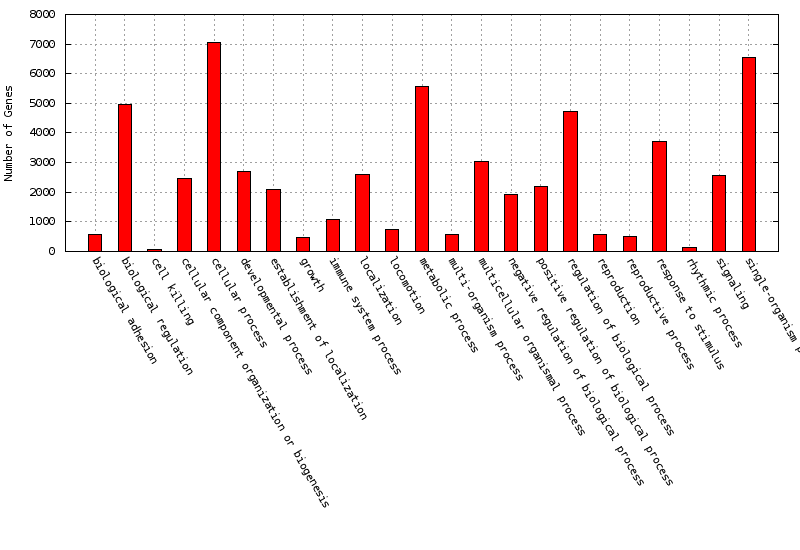


H5N8 7 dpi

H5N1 7 dpi

**Supplementary Fig. 5. Pathway for pattern recognition receptors recognizing viruses at 3 days post infection (dpi)**

Pathway analysis with IPA software allowed identification of pathways that were differentially expressed between H5N1- and H5N8- infected mice. Colour intensity indicates the degree of upregulation (red) or downregulation (green) relative to the mock-infected mice. Solid lines represent direct interactions and dashed lines indirect interactions.


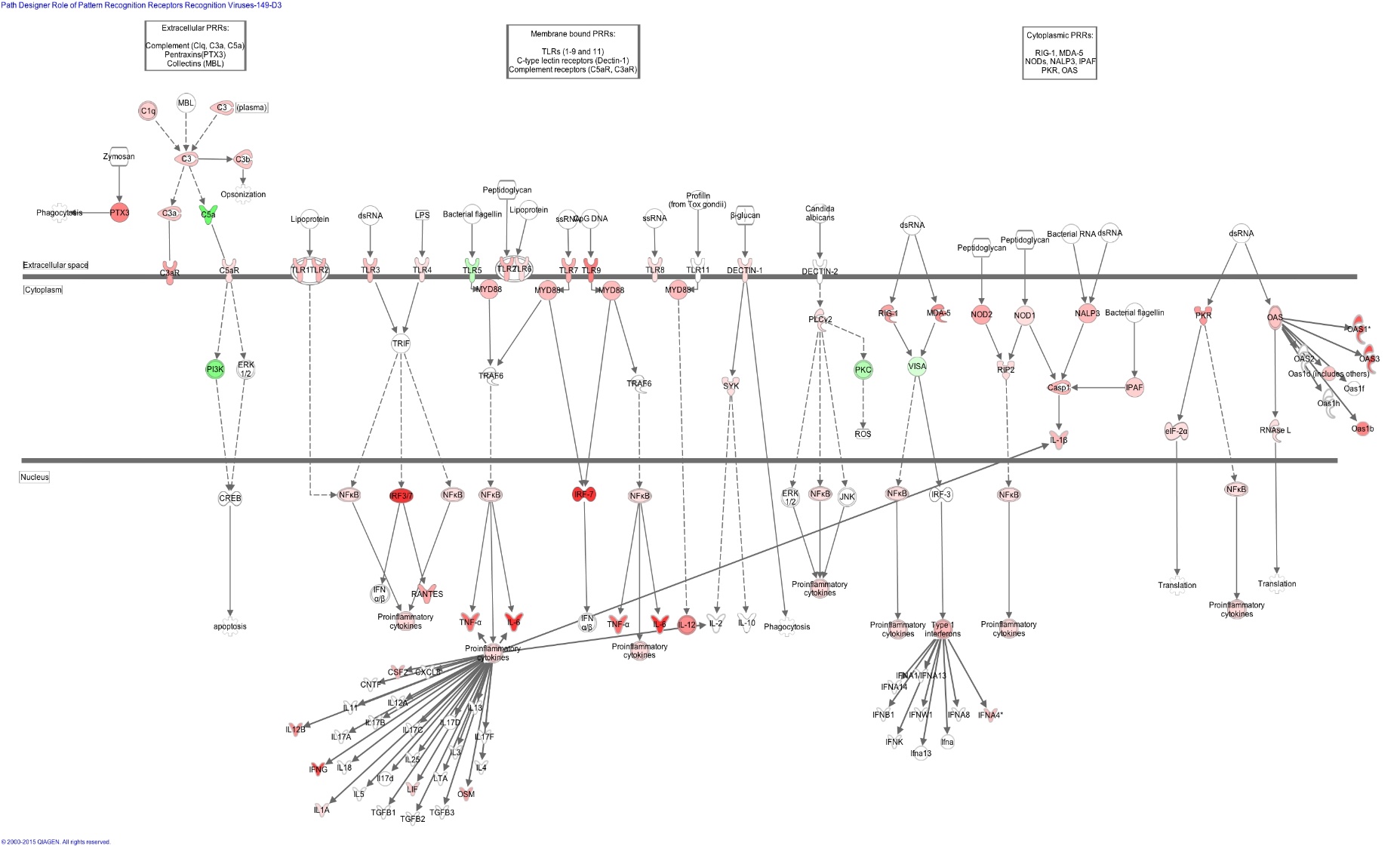


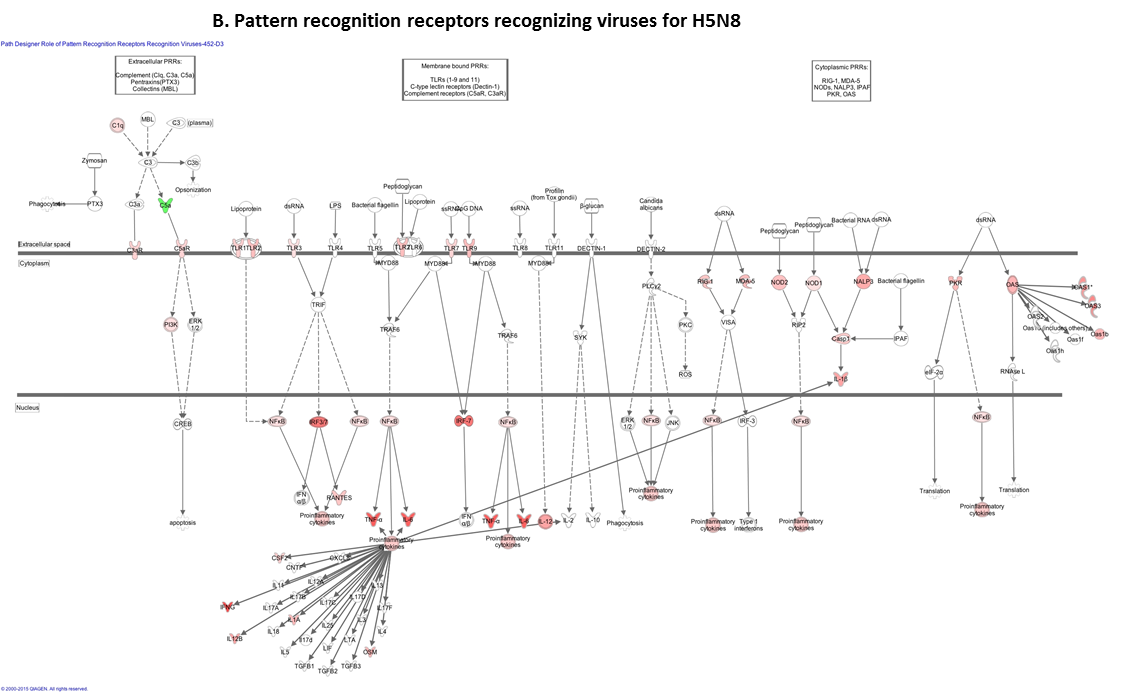


**Supplementary Fig. 6. Pathway for acute phase signalling network at 7 days post infection (dpi)**

Pathway analysis with IPA software allowed identification of pathways that were differentially expressed between H5N1- and H5N8- infected mice. Colour intensity indicates the degree of upregulation (red) or downregulation (green) relative to the mock-infected mice. Solid lines represent direct interactions and dashed lines indirect interactions.


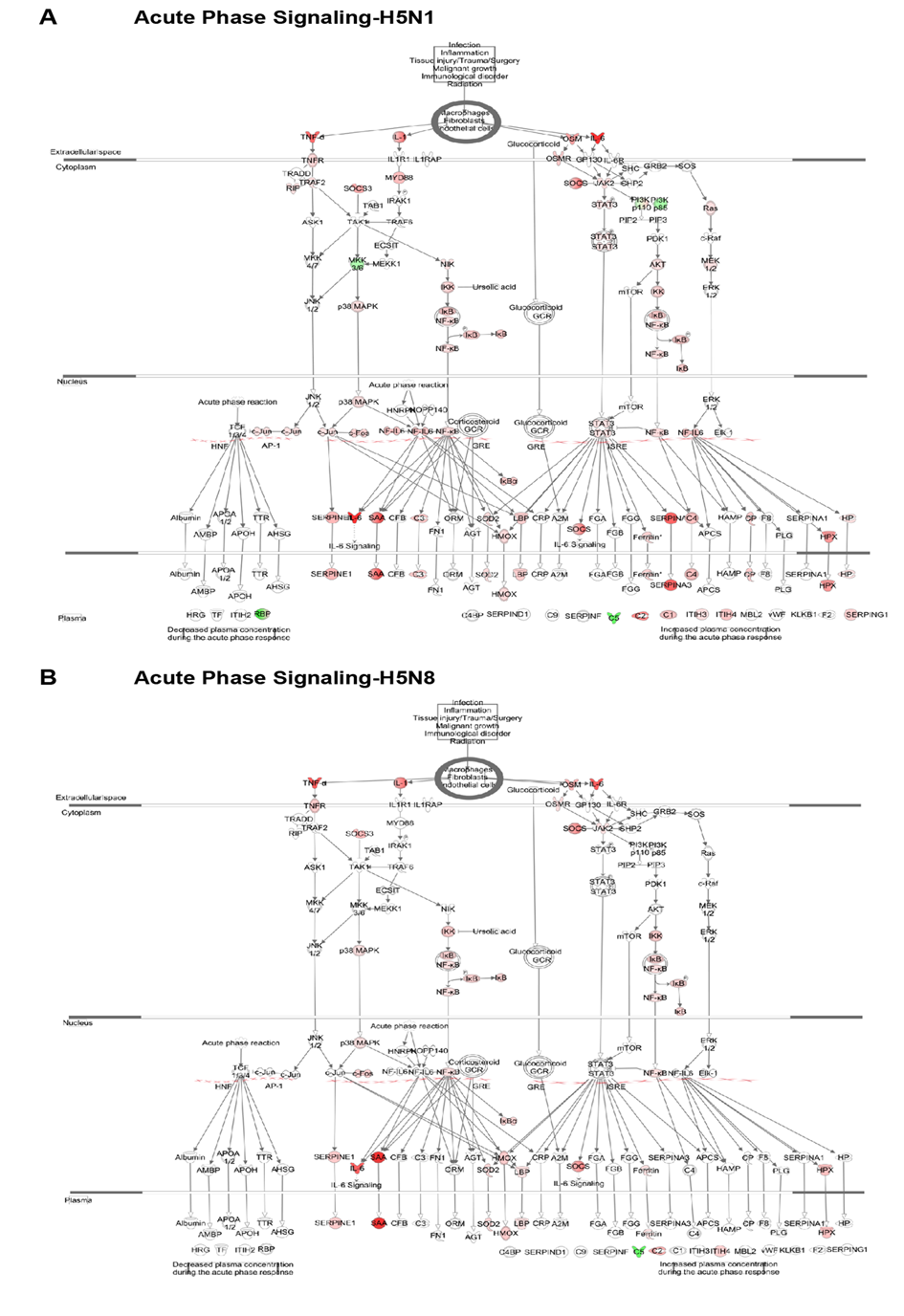


Supplementary Table 1. Significantly upregulated differentially expressed genes (DEGs) for H5N8-infected mice at 7 days post infection (dpi)

| **Gene Id** | **Gene Accession number** | **Gene Name** | **Description** |
| --- | --- | --- | --- |
| TBIG000958 | ENSMUSG00000026420.L01 | Il24 | Interleukin 24 [Source:MGI Symbol;Acc:MGI:2135548] |
| TBIG004042 | ENSMUSG00000020702 | Ccl1 | Chemokine (C-C motif) ligand 1 [Source:MGI Symbol;Acc:MGI:98258] |
| TBIG006146 | ENSMUSG00000094338 | Hist1h2bl | Histone cluster 1, H2bl [Source:MGI Symbol;Acc:MGI:2448403] |
| TBIG007042 | ENSMUSG00000096678 | Trav9-4 | T cell receptor alpha variable 9-4 [Source:MGI Symbol;Acc:MGI:3702135] |
| TBIG007051 | ENSMUSG00000076858 | Trav3-4 | T cell receptor alpha variable 3-4 [Source:MGI Symbol;Acc:MGI:2684925] |
| TBIG009198 | ENSMUSG00000022650 | Retnlb | Resistin like beta [Source:MGI Symbol;Acc:MGI:1888505] |
| TBIG015465 | ENSMUSG00000027718 | Il21 | Interleukin 21 [Source:MGI Symbol;Acc:MGI:1890474] |
| TBIG017575 | XLOC_017575 | TR22_MOUSE | Putative transposase element L1Md-A101/L1Md-A102/L1Md-A2 [Source:SWISS;Acc:P11260] |
| TBIG017857 | ENSMUSG00000062496 | 4930431F12Rik | RIKEN cDNA 4930431F12 gene [Source:MGI Symbol;Acc:MGI:1923033] |
| TBIG018257 | ENSMUSG00000006143.L01 | Upk3bl | Uroplakin 3B-like [Source:MGI Symbol;Acc:MGI:1916915] |
| TBIG019500 | XLOC_019500 | KV206_HUMAN | Ig kappa chain V-II region RPMI 6410 [Source:SWISS;Acc:P06310] |
| TBIG020099 | ENSMUSG00000094262 | Igkv4-62 | Immunoglobulin kappa variable 4-62 [Source:MGI Symbol;Acc:MGI:3643587] |
| TBIG020104 | XLOC_020104 | IGKV4-1 | Ig kappa chain V-IV region [Source:SWISS;Acc:P06312] |
| TBIG020105 | XLOC_020105 | Igk-V19-17 | Ig kappa chain V19-17 [Source:SWISS;Acc:P01633] |
| TBIG021651 | ENSMUSG00000025515.S02 | Muc2 | Mucin 2 [Source:MGI Symbol;Acc:MGI:1339364] |
| TBIG023626 | ENSMUSG00000079120 | AY761185 | cDNA sequence AY761185 [Source:MGI Symbol;Acc:MGI:3630303] |
| TBIG023693 | ENSMUSG00000031594.L01 | Fgl1 | Fibrinogen-like protein 1 [Source:MGI Symbol;Acc:MGI:102795] |
| TBIG026764 | XLOC_026764 | TR22_MOUSE | Putative transposase element L1Md-A101/L1Md-A102/L1Md-A2 [Source:SWISS;Acc:P11260] |

Supplementary Table 2. Significantly upregulated differentially expressed genes (DEGs) for H5N1-infected mice at 7 days post infection (dpi)

| **Gene Id** | **Gene Accession number** | **Gene Name** | **Description** |
| --- | --- | --- | --- |
| TBIG000958 | ENSMUSG00000026420.L01 | Il24 | Interleukin 24 [Source:MGI Symbol;Acc:MGI:2135548] |
| TBIG004042 | ENSMUSG00000020702 | Ccl1 | Chemokine (C-C motif) ligand 1 [Source:MGI Symbol;Acc:MGI:98258] |
| TBIG006146 | ENSMUSG00000094338 | Hist1h2bl | Histone cluster 1, H2bl [Source:MGI Symbol;Acc:MGI:2448403] |
| TBIG007051 | ENSMUSG00000076858 | Trav3-4 | T cell receptor alpha variable 3-4 [Source:MGI Symbol;Acc:MGI:2684925] |
| TBIG007634 | ENSMUSG00000022156 | Gzme | Granzyme E [Source:MGI Symbol;Acc:MGI:109265] |
| TBIG007635 | ENSMUSG00000059256 | Gzmd | Granzyme D [Source:MGI Symbol;Acc:MGI:109255] |
| TBIG007638 | ENSMUSG00000015441 | Gzmf | Granzyme F [Source:MGI Symbol;Acc:MGI:109254] |
| TBIG007931 | XLOC_007931 | Pol | Retrovirus-related Pol polyprotein LINE-1 [Source:SWISS;Acc:P11369] |
| TBIG008885 | XLOC_008885 | pol | Pol polyprotein [Source:SWISS;Acc:P21414] |
| TBIG009339 | ENSMUSG00000043050 | Tnp2 | Transition protein 2 [Source:MGI Symbol;Acc:MGI:98785] |
| TBIG010612 | XLOC_010612 | BTN3A3 | Butyrophilin subfamily 3 member A3 [Source:SWISS;Acc:O00478] |
| TBIG015959 | XLOC_015959 | TR22_MOUSE | Putative transposase element L1Md-A101/L1Md-A102/L1Md-A2 [Source:SWISS;Acc:P11260] |
| TBIG015465 | ENSMUSG00000027718 | Il21 | Interleukin 21 [Source:MGI Symbol;Acc:MGI:1890474] |
| TBIG015973 | XLOC_015973 | Pol | Retrovirus-related Pol polyprotein LINE-1 [Source:SWISS;Acc:P11369] |
| TBIG015092 | ENSMUSG00000042212 | Sprr2d | Small proline-rich protein 2D [Source:MGI Symbol;Acc:MGI:1330347] |
| TBIG015095 | ENSMUSG00000046203.L01 | Sprr2g | Small proline-rich protein 2G [Source:MGI Symbol;Acc:MGI:1330348] |
| TBIG017575 | XLOC_017575 | TR22_MOUSE | Putative transposase element L1Md-A101/L1Md-A102/L1Md-A2 [Source:SWISS;Acc:P11260] |
| TBIG017621 | XLOC_017621 | Nedd4 | E3 ubiquitin-protein ligase NEDD4 [Source:SWISS;Acc:P46935] |
| TBIG017857 | ENSMUSG00000062496 | 4930431F12Rik | RIKEN cDNA 4930431F12 gene [Source:MGI Symbol;Acc:MGI:1923033] |
| TBIG018257 | ENSMUSG00000006143.L01 | Upk3bl | Uroplakin 3B-like [Source:MGI Symbol;Acc:MGI:1916915] |
| TBIG020099 | ENSMUSG00000094262 | Igkv4-62 | Immunoglobulin kappa variable 4-62 [Source:MGI Symbol;Acc:MGI:3643587] |
| TBIG020105 | XLOC_020105 | Igk-V19-17 | Ig kappa chain V19-17 [Source:SWISS;Acc:P01633] |
| TBIG020234 | XLOC_020234 | Pol | Retrovirus-related Pol polyprotein LINE-1 [Source:SWISS;Acc:P11369] |
| TBIG022177 | ENSMUSG00000040017 | Saa4 | Serum amyloid A 4 [Source:MGI Symbol;Acc:MGI:98224] |
| TBIG023693 | ENSMUSG00000031594.L01 | Fgl1 | Fibrinogen-like protein 1 [Source:MGI Symbol;Acc:MGI:102795] |
| TBIG026244 | ENSMUSG00000031163 | Glod5 | Glyoxalase domain containing 5 [Source:MGI Symbol;Acc:MGI:1917074] |
| TBIG026764 | XLOC_026764 | TR22_MOUSE | Putative transposase element L1Md-A101/L1Md-A102/L1Md-A2 [Source:SWISS;Acc:P11260] |

Supplementary Table 3. Top 10 Gene ontology (GO) analysis for H5N1-infected mice

| **Days** | **No.** | **ACC** | **p-value** | **Category** | **# Transcripts** | **Definition** |
| --- | --- | --- | --- | --- | --- | --- |
| **1 dpi** | 1 | [GO:0061061](../../20150701%20H5N8%20paper/H5N8%20REVISION%2020150706/2015/%ED%85%8C%EB%9D%BC%EC%A0%A0%20H5N8%20RNAseq%20analysis/CHU-Human-Transcriptome-2015-01_V1_Quant/GO/biological_process/GO_0061061.html#S0019_S0020_S0021-S0004_S0005_S0006) | 1.74E-11 | anatomical structure development | 32 | muscle structure development |
| 2 | [GO:0055001](../../20150701%20H5N8%20paper/H5N8%20REVISION%2020150706/2015/%ED%85%8C%EB%9D%BC%EC%A0%A0%20H5N8%20RNAseq%20analysis/CHU-Human-Transcriptome-2015-01_V1_Quant/GO/biological_process/GO_0055001.html#S0019_S0020_S0021-S0004_S0005_S0006) | 5.30E-09 | muscle cell development | 16 | muscle cell development |
| 3 | [GO:0006950](../../20150701%20H5N8%20paper/H5N8%20REVISION%2020150706/2015/%ED%85%8C%EB%9D%BC%EC%A0%A0%20H5N8%20RNAseq%20analysis/CHU-Human-Transcriptome-2015-01_V1_Quant/GO/biological_process/GO_0006950.html#S0019_S0020_S0021-S0004_S0005_S0006) | 6.08E-09 | biological_process | 76 | response to stress |
| 4 | [GO:0051146](../../20150701%20H5N8%20paper/H5N8%20REVISION%2020150706/2015/%ED%85%8C%EB%9D%BC%EC%A0%A0%20H5N8%20RNAseq%20analysis/CHU-Human-Transcriptome-2015-01_V1_Quant/GO/biological_process/GO_0051146.html#S0019_S0020_S0021-S0004_S0005_S0006) | 7.54E-09 | striated muscle cell differentiation | 19 | striated muscle cell differentiation |
| 5 | [GO:0048878](../../20150701%20H5N8%20paper/H5N8%20REVISION%2020150706/2015/%ED%85%8C%EB%9D%BC%EC%A0%A0%20H5N8%20RNAseq%20analysis/CHU-Human-Transcriptome-2015-01_V1_Quant/GO/biological_process/GO_0048878.html#S0019_S0020_S0021-S0004_S0005_S0006) | 1.01E-08 | biological regulation | 35 | chemical homeostasis |
| 6 | [GO:0003012](../../20150701%20H5N8%20paper/H5N8%20REVISION%2020150706/2015/%ED%85%8C%EB%9D%BC%EC%A0%A0%20H5N8%20RNAseq%20analysis/CHU-Human-Transcriptome-2015-01_V1_Quant/GO/biological_process/GO_0003012.html#S0019_S0020_S0021-S0004_S0005_S0006) | 1.31E-08 | single-organism process | 19 | muscle system process |
| 7 | [GO:0055002](../../20150701%20H5N8%20paper/H5N8%20REVISION%2020150706/2015/%ED%85%8C%EB%9D%BC%EC%A0%A0%20H5N8%20RNAseq%20analysis/CHU-Human-Transcriptome-2015-01_V1_Quant/GO/biological_process/GO_0055002.html#S0019_S0020_S0021-S0004_S0005_S0006) | 1.31E-08 | striated muscle cell development | 15 | striated muscle cell development |
| 8 | [GO:0006936](../../20150701%20H5N8%20paper/H5N8%20REVISION%2020150706/2015/%ED%85%8C%EB%9D%BC%EC%A0%A0%20H5N8%20RNAseq%20analysis/CHU-Human-Transcriptome-2015-01_V1_Quant/GO/biological_process/GO_0006936.html#S0019_S0020_S0021-S0004_S0005_S0006) | 1.99E-08 | single-organism process | 17 | muscle contraction |
| 9 | [GO:0042692](../../20150701%20H5N8%20paper/H5N8%20REVISION%2020150706/2015/%ED%85%8C%EB%9D%BC%EC%A0%A0%20H5N8%20RNAseq%20analysis/CHU-Human-Transcriptome-2015-01_V1_Quant/GO/biological_process/GO_0042692.html#S0019_S0020_S0021-S0004_S0005_S0006) | 2.57E-08 | cellular developmental process | 21 | muscle cell differentiation |
| 10 | [GO:0014706](../../20150701%20H5N8%20paper/H5N8%20REVISION%2020150706/2015/%ED%85%8C%EB%9D%BC%EC%A0%A0%20H5N8%20RNAseq%20analysis/CHU-Human-Transcriptome-2015-01_V1_Quant/GO/biological_process/GO_0014706.html#S0019_S0020_S0021-S0004_S0005_S0006) | 3.66E-08 | anatomical structure development | 22 | striated muscle tissue development |
| **3 dpi** | 1 | [GO:0006955](../../20150701%20H5N8%20paper/H5N8%20REVISION%2020150706/2015/%ED%85%8C%EB%9D%BC%EC%A0%A0%20H5N8%20RNAseq%20analysis/CHU-Human-Transcriptome-2015-01_V1_Quant/GO/biological_process/GO_0006955.html#S0019_S0020_S0021-S0010_S0011_S0012) | 7.47E-84 | biological_process | 469 | immune response |
| 2 | [GO:0002376](../../20150701%20H5N8%20paper/H5N8%20REVISION%2020150706/2015/%ED%85%8C%EB%9D%BC%EC%A0%A0%20H5N8%20RNAseq%20analysis/CHU-Human-Transcriptome-2015-01_V1_Quant/GO/biological_process/GO_0002376.html#S0019_S0020_S0021-S0010_S0011_S0012) | 1.10E-81 | immune system process | 798 | immune system process |
| 3 | [GO:0006952](../../20150701%20H5N8%20paper/H5N8%20REVISION%2020150706/2015/%ED%85%8C%EB%9D%BC%EC%A0%A0%20H5N8%20RNAseq%20analysis/CHU-Human-Transcriptome-2015-01_V1_Quant/GO/biological_process/GO_0006952.html#S0019_S0020_S0021-S0010_S0011_S0012) | 2.42E-60 | biological_process | 464 | defense response |
| 4 | [GO:0002682](../../20150701%20H5N8%20paper/H5N8%20REVISION%2020150706/2015/%ED%85%8C%EB%9D%BC%EC%A0%A0%20H5N8%20RNAseq%20analysis/CHU-Human-Transcriptome-2015-01_V1_Quant/GO/biological_process/GO_0002682.html#S0019_S0020_S0021-S0010_S0011_S0012) | 9.68E-59 | regulation of biological process | 435 | regulation of immune system process |
| 5 | [GO:0009607](../../20150701%20H5N8%20paper/H5N8%20REVISION%2020150706/2015/%ED%85%8C%EB%9D%BC%EC%A0%A0%20H5N8%20RNAseq%20analysis/CHU-Human-Transcriptome-2015-01_V1_Quant/GO/biological_process/GO_0009607.html#S0019_S0020_S0021-S0010_S0011_S0012) | 4.45E-47 | response to biotic stimulus | 334 | response to biotic stimulus |
| 6 | [GO:0002684](../../20150701%20H5N8%20paper/H5N8%20REVISION%2020150706/2015/%ED%85%8C%EB%9D%BC%EC%A0%A0%20H5N8%20RNAseq%20analysis/CHU-Human-Transcriptome-2015-01_V1_Quant/GO/biological_process/GO_0002684.html#S0019_S0020_S0021-S0010_S0011_S0012) | 3.93E-46 | regulation of biological process | 290 | positive regulation of immune system process |
| 7 | [GO:0051707](../../20150701%20H5N8%20paper/H5N8%20REVISION%2020150706/2015/%ED%85%8C%EB%9D%BC%EC%A0%A0%20H5N8%20RNAseq%20analysis/CHU-Human-Transcriptome-2015-01_V1_Quant/GO/biological_process/GO_0051707.html#S0019_S0020_S0021-S0010_S0011_S0012) | 4.13E-46 | multi-organism process | 319 | response to other organism |
| 8 | [GO:0002252](../../20150701%20H5N8%20paper/H5N8%20REVISION%2020150706/2015/%ED%85%8C%EB%9D%BC%EC%A0%A0%20H5N8%20RNAseq%20analysis/CHU-Human-Transcriptome-2015-01_V1_Quant/GO/biological_process/GO_0002252.html#S0019_S0020_S0021-S0010_S0011_S0012) | 4.64E-46 | immune system process | 270 | immune effector process |
| 9 | [GO:0050776](../../20150701%20H5N8%20paper/H5N8%20REVISION%2020150706/2015/%ED%85%8C%EB%9D%BC%EC%A0%A0%20H5N8%20RNAseq%20analysis/CHU-Human-Transcriptome-2015-01_V1_Quant/GO/biological_process/GO_0050776.html#S0019_S0020_S0021-S0010_S0011_S0012) | 1.94E-44 | response to stimulus | 255 | regulation of immune response |
| 10 | [GO:0045087](../../20150701%20H5N8%20paper/H5N8%20REVISION%2020150706/2015/%ED%85%8C%EB%9D%BC%EC%A0%A0%20H5N8%20RNAseq%20analysis/CHU-Human-Transcriptome-2015-01_V1_Quant/GO/biological_process/GO_0045087.html#S0019_S0020_S0021-S0010_S0011_S0012) | 1.32E-42 | innate immune response | 221 | innate immune response |
| **7 dpi** | 1 | [GO:0002376](../../20150701%20H5N8%20paper/H5N8%20REVISION%2020150706/2015/%ED%85%8C%EB%9D%BC%EC%A0%A0%20H5N8%20RNAseq%20analysis/CHU-Human-Transcriptome-2015-01_V1_Quant/GO/biological_process/GO_0002376.html#S0019_S0020_S0021-S0016_S0017_S0018) | 2.05E-49 | immune system process | 1084 | immune system process |
| 2 | [GO:0006955](../../20150701%20H5N8%20paper/H5N8%20REVISION%2020150706/2015/%ED%85%8C%EB%9D%BC%EC%A0%A0%20H5N8%20RNAseq%20analysis/CHU-Human-Transcriptome-2015-01_V1_Quant/GO/biological_process/GO_0006955.html#S0019_S0020_S0021-S0016_S0017_S0018) | 1.69E-48 | biological_process | 586 | immune response |
| 3 | [GO:0048583](../../20150701%20H5N8%20paper/H5N8%20REVISION%2020150706/2015/%ED%85%8C%EB%9D%BC%EC%A0%A0%20H5N8%20RNAseq%20analysis/CHU-Human-Transcriptome-2015-01_V1_Quant/GO/biological_process/GO_0048583.html#S0019_S0020_S0021-S0016_S0017_S0018) | 1.11E-41 | response to stimulus | 1636 | regulation of response to stimulus |
| 4 | [GO:0048584](../../20150701%20H5N8%20paper/H5N8%20REVISION%2020150706/2015/%ED%85%8C%EB%9D%BC%EC%A0%A0%20H5N8%20RNAseq%20analysis/CHU-Human-Transcriptome-2015-01_V1_Quant/GO/biological_process/GO_0048584.html#S0019_S0020_S0021-S0016_S0017_S0018) | 1.13E-35 | response to stimulus | 850 | positive regulation of response to stimulus |
| 5 | [GO:0048518](../../20150701%20H5N8%20paper/H5N8%20REVISION%2020150706/2015/%ED%85%8C%EB%9D%BC%EC%A0%A0%20H5N8%20RNAseq%20analysis/CHU-Human-Transcriptome-2015-01_V1_Quant/GO/biological_process/GO_0048518.html#S0019_S0020_S0021-S0016_S0017_S0018) | 3.18E-33 | regulation of biological process | 2201 | positive regulation of biological process |
| 6 | [GO:0002682](../../20150701%20H5N8%20paper/H5N8%20REVISION%2020150706/2015/%ED%85%8C%EB%9D%BC%EC%A0%A0%20H5N8%20RNAseq%20analysis/CHU-Human-Transcriptome-2015-01_V1_Quant/GO/biological_process/GO_0002682.html#S0019_S0020_S0021-S0016_S0017_S0018) | 5.37E-33 | regulation of biological process | 560 | regulation of immune system process |
| 7 | [GO:0002684](../../20150701%20H5N8%20paper/H5N8%20REVISION%2020150706/2015/%ED%85%8C%EB%9D%BC%EC%A0%A0%20H5N8%20RNAseq%20analysis/CHU-Human-Transcriptome-2015-01_V1_Quant/GO/biological_process/GO_0002684.html#S0019_S0020_S0021-S0016_S0017_S0018) | 1.10E-32 | regulation of biological process | 379 | positive regulation of immune system process |
| 8 | [GO:0009966](../../20150701%20H5N8%20paper/H5N8%20REVISION%2020150706/2015/%ED%85%8C%EB%9D%BC%EC%A0%A0%20H5N8%20RNAseq%20analysis/CHU-Human-Transcriptome-2015-01_V1_Quant/GO/biological_process/GO_0009966.html#S0019_S0020_S0021-S0016_S0017_S0018) | 3.57E-32 | cellular response to stimulus | 1310 | regulation of signal transduction |
| 9 | [GO:0006950](../../20150701%20H5N8%20paper/H5N8%20REVISION%2020150706/2015/%ED%85%8C%EB%9D%BC%EC%A0%A0%20H5N8%20RNAseq%20analysis/CHU-Human-Transcriptome-2015-01_V1_Quant/GO/biological_process/GO_0006950.html#S0019_S0020_S0021-S0016_S0017_S0018) | 7.11E-32 | biological_process | 1530 | response to stress |
| 10 | [GO:0010646](../../20150701%20H5N8%20paper/H5N8%20REVISION%2020150706/2015/%ED%85%8C%EB%9D%BC%EC%A0%A0%20H5N8%20RNAseq%20analysis/CHU-Human-Transcriptome-2015-01_V1_Quant/GO/biological_process/GO_0010646.html#S0019_S0020_S0021-S0016_S0017_S0018) | 6.81E-31 | regulation of cellular process | 1456 | regulation of cell communication |

Supplementary Table 4. Top 10 Gene ontology (GO) analysis for H5N8-infected mice

| **Days** | **No.** | **ACC** | **p-value** | **Category** | **# Transcripts** | **Definition** |
| --- | --- | --- | --- | --- | --- | --- |
| 1 dpi | 1 | [GO:0043588](../../20150701%20H5N8%20paper/H5N8%20REVISION%2020150706/2015/%ED%85%8C%EB%9D%BC%EC%A0%A0%20H5N8%20RNAseq%20analysis/CHU-Human-Transcriptome-2015-01_V1_Quant/GO/biological_process/GO_0043588.html#S0019_S0020_S0021-S0001_S0002_S0003) | 3.35E-18 | system development | 90 | skin development |
| 2 | [GO:0008544](../../20150701%20H5N8%20paper/H5N8%20REVISION%2020150706/2015/%ED%85%8C%EB%9D%BC%EC%A0%A0%20H5N8%20RNAseq%20analysis/CHU-Human-Transcriptome-2015-01_V1_Quant/GO/biological_process/GO_0008544.html#S0019_S0020_S0021-S0001_S0002_S0003) | 6.19E-18 | system development | 80 | epidermis development |
| 3 | [GO:0009888](../../20150701%20H5N8%20paper/H5N8%20REVISION%2020150706/2015/%ED%85%8C%EB%9D%BC%EC%A0%A0%20H5N8%20RNAseq%20analysis/CHU-Human-Transcriptome-2015-01_V1_Quant/GO/biological_process/GO_0009888.html#S0019_S0020_S0021-S0001_S0002_S0003) | 5.31E-16 | developmental process | 286 | tissue development |
| 4 | [GO:0003012](../../20150701%20H5N8%20paper/H5N8%20REVISION%2020150706/2015/%ED%85%8C%EB%9D%BC%EC%A0%A0%20H5N8%20RNAseq%20analysis/CHU-Human-Transcriptome-2015-01_V1_Quant/GO/biological_process/GO_0003012.html#S0019_S0020_S0021-S0001_S0002_S0003) | 3.98E-15 | single-organism process | 73 | muscle system process |
| 5 | [GO:0043086](../../20150701%20H5N8%20paper/H5N8%20REVISION%2020150706/2015/%ED%85%8C%EB%9D%BC%EC%A0%A0%20H5N8%20RNAseq%20analysis/CHU-Human-Transcriptome-2015-01_V1_Quant/GO/biological_process/GO_0043086.html#S0019_S0020_S0021-S0001_S0002_S0003) | 6.34E-15 | biological regulation | 127 | negative regulation of catalytic activity |
| 6 | [GO:0006950](../../20150701%20H5N8%20paper/H5N8%20REVISION%2020150706/2015/%ED%85%8C%EB%9D%BC%EC%A0%A0%20H5N8%20RNAseq%20analysis/CHU-Human-Transcriptome-2015-01_V1_Quant/GO/biological_process/GO_0006950.html#S0019_S0020_S0021-S0001_S0002_S0003) | 6.64E-15 | biological_process | 392 | response to stress |
| 7 | [GO:0048513](../../20150701%20H5N8%20paper/H5N8%20REVISION%2020150706/2015/%ED%85%8C%EB%9D%BC%EC%A0%A0%20H5N8%20RNAseq%20analysis/CHU-Human-Transcriptome-2015-01_V1_Quant/GO/biological_process/GO_0048513.html#S0019_S0020_S0021-S0001_S0002_S0003) | 2.39E-14 | system development | 448 | organ development |
| 8 | [GO:0044767](../../20150701%20H5N8%20paper/H5N8%20REVISION%2020150706/2015/%ED%85%8C%EB%9D%BC%EC%A0%A0%20H5N8%20RNAseq%20analysis/CHU-Human-Transcriptome-2015-01_V1_Quant/GO/biological_process/GO_0044767.html#S0019_S0020_S0021-S0001_S0002_S0003) | 1.22E-13 | single-organism process | 698 | single-organism developmental process |
| 9 | [GO:0006936](../../20150701%20H5N8%20paper/H5N8%20REVISION%2020150706/2015/%ED%85%8C%EB%9D%BC%EC%A0%A0%20H5N8%20RNAseq%20analysis/CHU-Human-Transcriptome-2015-01_V1_Quant/GO/biological_process/GO_0006936.html#S0019_S0020_S0021-S0001_S0002_S0003) | 2.12E-13 | single-organism process | 61 | muscle contraction |
| 10 | [GO:0032502](../../20150701%20H5N8%20paper/H5N8%20REVISION%2020150706/2015/%ED%85%8C%EB%9D%BC%EC%A0%A0%20H5N8%20RNAseq%20analysis/CHU-Human-Transcriptome-2015-01_V1_Quant/GO/biological_process/GO_0032502.html#S0019_S0020_S0021-S0001_S0002_S0003) | 2.64E-13 | biological_process | 698 | developmental process |
| 3 dpi | 1 | [GO:0006955](../../20150701%20H5N8%20paper/H5N8%20REVISION%2020150706/2015/%ED%85%8C%EB%9D%BC%EC%A0%A0%20H5N8%20RNAseq%20analysis/CHU-Human-Transcriptome-2015-01_V1_Quant/GO/biological_process/GO_0006955.html#S0019_S0020_S0021-S0007_S0008_S0009) | 1.99E-91 | biological_process | 155 | immune response |
| 2 | [GO:0006952](../../20150701%20H5N8%20paper/H5N8%20REVISION%2020150706/2015/%ED%85%8C%EB%9D%BC%EC%A0%A0%20H5N8%20RNAseq%20analysis/CHU-Human-Transcriptome-2015-01_V1_Quant/GO/biological_process/GO_0006952.html#S0019_S0020_S0021-S0007_S0008_S0009) | 3.20E-84 | biological_process | 155 | defense response |
| 3 | [GO:0002376](../../20150701%20H5N8%20paper/H5N8%20REVISION%2020150706/2015/%ED%85%8C%EB%9D%BC%EC%A0%A0%20H5N8%20RNAseq%20analysis/CHU-Human-Transcriptome-2015-01_V1_Quant/GO/biological_process/GO_0002376.html#S0019_S0020_S0021-S0007_S0008_S0009) | 8.02E-78 | immune system process | 195 | immune system process |
| 4 | [GO:0051707](../../20150701%20H5N8%20paper/H5N8%20REVISION%2020150706/2015/%ED%85%8C%EB%9D%BC%EC%A0%A0%20H5N8%20RNAseq%20analysis/CHU-Human-Transcriptome-2015-01_V1_Quant/GO/biological_process/GO_0051707.html#S0019_S0020_S0021-S0007_S0008_S0009) | 1.89E-72 | multi-organism process | 120 | response to other organism |
| 5 | [GO:0009607](../../20150701%20H5N8%20paper/H5N8%20REVISION%2020150706/2015/%ED%85%8C%EB%9D%BC%EC%A0%A0%20H5N8%20RNAseq%20analysis/CHU-Human-Transcriptome-2015-01_V1_Quant/GO/biological_process/GO_0009607.html#S0019_S0020_S0021-S0007_S0008_S0009) | 1.42E-71 | response to biotic stimulus | 122 | response to biotic stimulus |
| 6 | [GO:0045087](../../20150701%20H5N8%20paper/H5N8%20REVISION%2020150706/2015/%ED%85%8C%EB%9D%BC%EC%A0%A0%20H5N8%20RNAseq%20analysis/CHU-Human-Transcriptome-2015-01_V1_Quant/GO/biological_process/GO_0045087.html#S0019_S0020_S0021-S0007_S0008_S0009) | 2.63E-67 | innate immune response | 95 | innate immune response |
| 7 | [GO:0051704](../../20150701%20H5N8%20paper/H5N8%20REVISION%2020150706/2015/%ED%85%8C%EB%9D%BC%EC%A0%A0%20H5N8%20RNAseq%20analysis/CHU-Human-Transcriptome-2015-01_V1_Quant/GO/biological_process/GO_0051704.html#S0019_S0020_S0021-S0007_S0008_S0009) | 1.03E-57 | biological_process | 130 | multi-organism process |
| 8 | [GO:0098542](../../20150701%20H5N8%20paper/H5N8%20REVISION%2020150706/2015/%ED%85%8C%EB%9D%BC%EC%A0%A0%20H5N8%20RNAseq%20analysis/CHU-Human-Transcriptome-2015-01_V1_Quant/GO/biological_process/GO_0098542.html#S0019_S0020_S0021-S0007_S0008_S0009) | 1.53E-55 | response to other organism | 78 | defense response to other organism |
| 9 | [GO:0002252](../../20150701%20H5N8%20paper/H5N8%20REVISION%2020150706/2015/%ED%85%8C%EB%9D%BC%EC%A0%A0%20H5N8%20RNAseq%20analysis/CHU-Human-Transcriptome-2015-01_V1_Quant/GO/biological_process/GO_0002252.html#S0019_S0020_S0021-S0007_S0008_S0009) | 3.54E-51 | immune system process | 90 | immune effector process |
| 10 | [GO:0009615](../../20150701%20H5N8%20paper/H5N8%20REVISION%2020150706/2015/%ED%85%8C%EB%9D%BC%EC%A0%A0%20H5N8%20RNAseq%20analysis/CHU-Human-Transcriptome-2015-01_V1_Quant/GO/biological_process/GO_0009615.html#S0019_S0020_S0021-S0007_S0008_S0009) | 8.87E-46 | multi-organism process | 61 | response to virus |
| 7 dpi | 1 | [GO:0002376](../../20150701%20H5N8%20paper/H5N8%20REVISION%2020150706/2015/%ED%85%8C%EB%9D%BC%EC%A0%A0%20H5N8%20RNAseq%20analysis/CHU-Human-Transcriptome-2015-01_V1_Quant/GO/biological_process/GO_0002376.html#S0019_S0020_S0021-S0013_S0014_S0015) | 1.97E-84 | immune system process | 727 | immune system process |
| 2 | [GO:0006955](../../20150701%20H5N8%20paper/H5N8%20REVISION%2020150706/2015/%ED%85%8C%EB%9D%BC%EC%A0%A0%20H5N8%20RNAseq%20analysis/CHU-Human-Transcriptome-2015-01_V1_Quant/GO/biological_process/GO_0006955.html#S0019_S0020_S0021-S0013_S0014_S0015) | 1.43E-82 | biological_process | 429 | immune response |
| 3 | [GO:0006952](../../20150701%20H5N8%20paper/H5N8%20REVISION%2020150706/2015/%ED%85%8C%EB%9D%BC%EC%A0%A0%20H5N8%20RNAseq%20analysis/CHU-Human-Transcriptome-2015-01_V1_Quant/GO/biological_process/GO_0006952.html#S0019_S0020_S0021-S0013_S0014_S0015) | 3.69E-62 | biological_process | 426 | defense response |
| 4 | [GO:0002682](../../20150701%20H5N8%20paper/H5N8%20REVISION%2020150706/2015/%ED%85%8C%EB%9D%BC%EC%A0%A0%20H5N8%20RNAseq%20analysis/CHU-Human-Transcriptome-2015-01_V1_Quant/GO/biological_process/GO_0002682.html#S0019_S0020_S0021-S0013_S0014_S0015) | 4.89E-60 | regulation of biological process | 399 | regulation of immune system process |
| 5 | [GO:0002684](../../20150701%20H5N8%20paper/H5N8%20REVISION%2020150706/2015/%ED%85%8C%EB%9D%BC%EC%A0%A0%20H5N8%20RNAseq%20analysis/CHU-Human-Transcriptome-2015-01_V1_Quant/GO/biological_process/GO_0002684.html#S0019_S0020_S0021-S0013_S0014_S0015) | 1.14E-52 | regulation of biological process | 277 | positive regulation of immune system process |
| 6 | [GO:0006950](../../20150701%20H5N8%20paper/H5N8%20REVISION%2020150706/2015/%ED%85%8C%EB%9D%BC%EC%A0%A0%20H5N8%20RNAseq%20analysis/CHU-Human-Transcriptome-2015-01_V1_Quant/GO/biological_process/GO_0006950.html#S0019_S0020_S0021-S0013_S0014_S0015) | 1.36E-46 | biological_process | 906 | response to stress |
| 7 | [GO:0002252](../../20150701%20H5N8%20paper/H5N8%20REVISION%2020150706/2015/%ED%85%8C%EB%9D%BC%EC%A0%A0%20H5N8%20RNAseq%20analysis/CHU-Human-Transcriptome-2015-01_V1_Quant/GO/biological_process/GO_0002252.html#S0019_S0020_S0021-S0013_S0014_S0015) | 4.27E-46 | immune system process | 248 | immune effector process |
| 8 | [GO:0050776](../../20150701%20H5N8%20paper/H5N8%20REVISION%2020150706/2015/%ED%85%8C%EB%9D%BC%EC%A0%A0%20H5N8%20RNAseq%20analysis/CHU-Human-Transcriptome-2015-01_V1_Quant/GO/biological_process/GO_0050776.html#S0019_S0020_S0021-S0013_S0014_S0015) | 1.97E-45 | response to stimulus | 236 | regulation of immune response |
| 9 | [GO:0045087](../../20150701%20H5N8%20paper/H5N8%20REVISION%2020150706/2015/%ED%85%8C%EB%9D%BC%EC%A0%A0%20H5N8%20RNAseq%20analysis/CHU-Human-Transcriptome-2015-01_V1_Quant/GO/biological_process/GO_0045087.html#S0019_S0020_S0021-S0013_S0014_S0015) | 3.90E-44 | innate immune response | 206 | innate immune response |
| 10 | [GO:0051707](../../20150701%20H5N8%20paper/H5N8%20REVISION%2020150706/2015/%ED%85%8C%EB%9D%BC%EC%A0%A0%20H5N8%20RNAseq%20analysis/CHU-Human-Transcriptome-2015-01_V1_Quant/GO/biological_process/GO_0051707.html#S0019_S0020_S0021-S0013_S0014_S0015) | 5.75E-44 | multi-organism process | 287 | response to other organism |
